# Hybrid Cardiac Rehabilitation Program in a Low-Resource Setting: A Randomized Clinical Trial

**DOI:** 10.1001/jamanetworkopen.2023.50301

**Published:** 2024-01-09

**Authors:** Pamela Seron, Maria Jose Oliveros, Gabriel Nasri Marzuca-Nassr, Gladys Morales, Claudia Román, Sergio Raúl Muñoz, Manuel Gálvez, Gonzalo Latin, Tania Marileo, Juan Pablo Molina, Rocío Navarro, Pablo Sepúlveda, Fernando Lanas, Nicolás Saavedra, Constanza Ulloa, Sherry L. Grace

**Affiliations:** 1 Facultad de Medicina, Departamento de Ciencias de la Rehabilitación, Universidad de La Frontera, Temuco, Chile; 2 Centro de Excelencia CIGES, Universidad de La Frontera, Temuco, Chile; 3 Facultad de Medicina, Departamento de Ciencias de la Rehabilitación, Universidad de La Frontera, Temuco, Chile; 4 Facultad de Medicina, Departamento de Salud Pública, Universidad de La Frontera, Temuco, Chile; 5 Facultad de Medicina, Escuela de Kinesiología, Pontificia Universidad Católica de Chile, Santiago, Chile; 6 Unidad de Kinesiología, Complejo Hospitalario San José, Santiago, Chile; 7 Servicio de Medicina Física y Rehabilitación, Hospital Clínico, Hospital San Borja Arriarán, Santiago, Chile; 8 Unidad de Rehabilitación Cardiaca, Hospital Regional de Antofagasta, Antofagasta, Chile; 9 Servicio de Medicina Física y Rehabilitación, Hospital San Juan de Dios, Santiago, Chile; 10 Servicio de Medicina Física y Rehabilitación, Hospital Clínico Universidad de Chile, Santiago, Chile; 11 Facultad de Medicina, Departamento de Medicina Interna, Universidad de La Frontera, Temuco, Chile; 12 Centro de Excelencia CIGES, Universidad de La Frontera, Temuco, Chile; 13 Facultad de Medicina, Departamento de Ciencias Básicas, Universidad de La Frontera, Temuco, Chile; 14 York University & University Health Network, University of Toronto, Toronto, Ontario, Canada

## Abstract

**Importance:**

While effective, cardiovascular rehabilitation (CR) as traditionally delivered is not well implemented in lower-resource settings.

**Objective:**

To test the noninferiority of hybrid CR compared with traditional CR in terms of cardiovascular events.

**Design, Setting, and Participants:**

This pragmatic, multicenter, parallel arm, open-label randomized clinical trial (the Hybrid Cardiac Rehabilitation Trial [HYCARET]) with blinded outcome assessment was conducted at 6 referral centers in Chile. Adults aged 18 years or older who had a cardiovascular event or procedure, no contraindications to exercise, and access to a mobile telephone were eligible and recruited between April 1, 2019, and March 15, 2020, with follow-up until July 29, 2021.

**Interventions:**

Participants were randomized 1:1 in permuted blocks to the experimental arm, which received 10 center-based supervised exercise sessions plus counseling in 4 to 6 weeks and then were supported at home via telephone calls and text messages through weeks 8 to 12, or the control arm, which received the standard CR of 18 to 22 sessions with exercises and education in 8 to 12 weeks.

**Main Outcomes and Measures:**

The primary outcome was cardiovascular events or mortality. Secondary outcomes were quality of life, return to work, and lifestyle behaviors measured with validated questionnaires; muscle strength and functional capacity, measured through physical tests; and program adherence and exercise-related adverse events, assessed using checklists.

**Results:**

A total of 191 participants were included (mean [SD] age, 58.74 [9.80] years; 145 [75.92%] male); 93 were assigned to hybrid CR and 98 to standard CR. At 1 year, events had occurred in 5 unique participants in the hybrid CR group (5.38%) and 9 in the standard CR group (9.18%). In the intention-to-treat analysis, the hybrid CR group had 3.80% (95% CI, –11.13% to 3.52%) fewer cardiovascular events than the standard CR group, and relative risk was 0.59 (95% CI, 0.20-1.68) for the primary outcome. In the per-protocol analysis at different levels of adherence to the intervention, all 95% CIs crossed the noninferiority boundary (eg, 20% adherence: absolute risk difference, −0.35% [95% CI, −7.56% to 6.85%]; 80% adherence: absolute risk difference, 3.30% [95% CI, −3.70% to 10.31%]). No between-group differences were found for secondary outcomes except adherence to supervised CR sessions (79.14% [736 of 930 supervised sessions] in the hybrid CR group vs 61.46% [1201 of 1954 sessions] in the standard CR group).

**Conclusions and Relevance:**

The results suggest that a hybrid CR program is noninferior to standard center-based CR in a low-resource setting, primarily in terms of recurrent cardiovascular events and potentially in terms of intermediate outcomes. Hybrid CR may induce superior adherence to supervised exercise. Clinical factors and patient preferences should inform CR model allocation.

**Trial Registration:**

ClinicalTrials.gov Identifier: NCT03881150

## Key Points

**Question:**

Is a hybrid model of cardiac rehabilitation (CR) noninferior to the traditional center-based model in terms of the occurrence of cardiovascular events?

**Findings:**

In this randomized clinical trial involving 191 patients with coronary artery disease, hybrid CR was not inferior to standard CR. Participants in the hybrid CR group had fewer cardiovascular events than participants in the standard CR group.

**Meaning:**

In low-resource settings, considering resource availability and patient preferences, a hybrid CR model may serve as an alternative intervention to a standard center-based program.

## Teaser

This randomized clinical trial of patients with coronary artery disease investigates the noninferiority of a hybrid center- and home-based cardiac rehabilitation program vs a traditional center-based program in terms of cardiac events.

## Introduction

Coronary heart disease remains highly prevalent worldwide, with 197.2 million cases annually and 182.0 million disability-adjusted life years.^1^ In southern Latin America, the prevalence is 1152.3 per 100 000 population, presenting a persistent concern.^1^

Individuals with coronary heart disease face a heightened risk of experiencing new cardiovascular events. This affects their daily functioning and societal participation and thus warrants rehabilitation. Cardiac rehabilitation (CR) is a recommended intervention to enhance prognosis.^2,3^ A Cochrane review indicated that CR was associated with reduced likelihood of a second heart attack by 7% and hospitalization risk by 23% compared with standard care and with significant improvements in health-related quality of life (HRQOL).^4^ Despite advancements in medical care, CR’s benefits, including cost-effectiveness, persist.^5,6^

Despite this evidence, CR remains underused globally, with only 55% of countries offering a CR program.^7^ Also, access to CR varies widely, with lower-resource regions, such as Latin America, experiencing lower rates.^8,9^

In situations in which CR is not widely implemented despite the proven efficacy and cost-effectiveness, such that the benefits are only reaped by a fraction of indicated patients, it is necessary to design alternative models or approaches to lowering the cost and increasing the coverage of CR. Studied approaches include unsupervised or home-based delivery, use of information and communication technology, delegation of tasks to lower-wage health care workers, and reduction of face-to-face sessions.^10^ Additionally, proposed strategies involve using more affordable exercise equipment and monitoring without telemetry.^10,11^

Cardiac rehabilitation is a complex intervention affording the opportunity to design models adapted to different contexts and, with the advent of the COVID-19 pandemic, to hybrid models in particular. In hybrid models, patients transition from a supervised, center-based phase as soon as it is appropriate to an unsupervised phase. Hybrid CR has been widely implemented and advocated.^12,13^ Thus, combining the International Council of Cardiovascular Prevention and Rehabilitation consensus recommendations for CR in low-resource settings^3^ with studies of alternative CR models (ie, home-based or technology-mediated CR, which to date has largely been applied in high-resource settings^14,15,16,17,18,19^), we developed a hybrid CR model.

The main objective of this randomized clinical trial was to evaluate whether the reduction in cardiovascular events (fatal and nonfatal) in patients receiving hybrid CR is not inferior to reductions in patients receiving standard supervised CR. A secondary aim was to compare hybrid CR with traditional supervised CR in terms of HRQOL, exercise capacity, muscle strength, heart-healthy behavior, return to work, cardiovascular risk factor control, adherence to CR, and exercise-related adverse events.

## Methods

The Hybrid Cardiac Rehabilitation Trial (HYCARET) (NCT03881150) was a noninferiority, pragmatic (ie, in a real-life context), multicenter, open-label, randomized clinical trial with 2 parallel arms and blinded end point assessment. This report has been prepared in accordance with the Consolidated Standards of Reporting Trials (CONSORT) Statement^20^ including extensions.^21,22,23^ In addition, mainly as a result of COVID-19 but also because of the political situation in Chile, we adhered to the CONSORT and SPIRIT Extension for RCTs Revised in Extenuating Circumstances (CONSERVE) Statement.^24^ The original study protocol (Supplement 1) and its subsequent amendments were approved by the corresponding scientific ethics committees at the participating centers (eTable 1 in Supplement 2) and have been described elsewhere.^25^ Written informed consent was obtained from all participants.

### Eligibility Criteria, Recruitment, and Assignment

Recruitment began April 1, 2019, and patients were approached consecutively until March 15, 2020, when accrual had to be stopped due to prevention and control measures related to COVID-19. Participants were included if they were aged 18 years or older; had coronary artery disease, including acute coronary syndrome or stable coronary vessel disease diagnosed by angiography or a stress test; had access to a mobile telephone; and provided written informed consent. The specific inclusion and exclusion criteria are listed in eMethods 1 in Supplement 2. Participants were consecutively recruited from 6 hospitals in Chile (eTable 1 in Supplement 2). Allocation to the hybrid CR or standard CR arm was stratified by center, with 1:1 permuted blocked randomization, and the concealment of the assignment was preserved through features available in REDCap.

### Interventions

Patients in the experimental arm participated in a hybrid CR program. It comprised 2 stages. Initially, supervised sessions were led by a physiotherapist at a center; after 10 sessions in 4 to 6 weeks, the program transitioned to a self-managed, home-based phase through weeks 8 to 12. The supervised exercise sessions involved aerobic exercise, muscular fitness, and flexibility exercise. Each session started with 5 to 10 minutes of warm-up, followed by 20 to 60 minutes of conditioning (in exercise bouts of at least 10 minutes) and finishing with 8 to 10 minutes of cooldown. Intensity was moderate (perceived exertion rated 12-13 on the Borg 6-20 scale^26^ and heart rate not higher than 60% of the heart rate reserve). Sessions were 2 to 3 times per week. In addition, the first stage included individual counseling delivered as a progressive dialogue between the physiotherapist and patient during face-to-face exercise sessions. This counseling was based on the theory of self-efficacy and supported by an accompanying manual titled *How Can I Live Better?*^27^

The second stage in the experimental arm was home based, and it consisted of telephone support through text messages twice a week and a total of 3 voice calls through weeks 8 to 12. The content of the text messages and voice calls was delivered by a trained health professional, adapted from a bank of suggestions,^28^ and focused on the benefits of physical activity and a healthy diet, recommended behaviors and strategies to achieve these, and medication adherence (eMethods 2 in Supplement 2).

The control group followed the standard face-to-face CR program, which included supervised aerobic and resistance training similar to that received by the experimental group. The program spanned 8 to 12 weeks with 18 to 22 sessions, based on patient needs and center availability. Additionally, there was 1 group education session covering various topics like physical activity, diet, tobacco use, and medication adherence that involved a multidisciplinary team.

### Outcomes and Measures

The primary outcome for which noninferiority was evaluated was cardiovascular events, defined as a composite of cardiovascular mortality (death by stroke, myocardial infarction, or heart failure) or new cardiovascular events (heart failure or hospitalization due to nonfatal stroke, nonfatal myocardial infarction, and need for revascularization surgery). To fully capture probable events, every 2 months, a research assistant contacted the participants to complete a brief health event survey. When applicable, for death adjudication including cause, the death certificate with associated medical documentation was collected. For probable hospitalization, a research assistant reviewed the clinical record with all associated tests and examination reports in each participating center. For all supporting documentation, an independent central adjudication committee (with 1 member being a cardiology specialist) blinded to participant allocation reviewed all information to make the final decision as to whether the event was definitive or rejected, specifying the final death cause or hospitalization diagnosis.

Secondary outcomes were assessed through questionnaires or physical measures by trained personnel blinded to participant allocation. Health-related quality of life was measured with the disease-specific HeartQoL instrument^29^ comprising 14 items covering 2 dimensions (physical and emotional). Total scores range from 0 to 3, where 0 is the best and 3 is the worst quality of life. In addition, the visual analog scale (0 to 100, with higher scores indicating better quality of life) of the EuroQol 5-dimension, 3-level measure was also administered.^30^ Functional exercise capacity was evaluated through the 6-minute walk test.^31^ Results were expressed in total distance walked in meters during the test. Muscle strength was evaluated through grip strength (kg) using a Jamar dynamometer (Patterson Medical) according to a standardized method.^32,33^ The International Physical Activity Questionnaire^34^ was administered to determine adherence to physical activity recommendations as defined by the World Health Organization.^35^ The Mediterranean Dietary Index for the Chilean population was used to assess adherence to diet recommendations; those who scored 5 or more points on a scale from 0 to 14 were considered adherent.^36,37^ For return to work, work status was queried at baseline and each point of follow-up; if the participants were paid workers at the time of the event that made them eligible for inclusion in CR, the date of their return to work was recorded, from which the time to return to work was calculated in days from baseline. Attendance at each supervised exercise session was recorded to calculate the adherence to CR in both arms as a percentage of the total sessions prescribed.^38^ Finally, exercise-related adverse events were recorded.

All outcomes were measured at baseline, at the end of the intervention, and at 6 months and 12 months of follow-up, with the last time of measurement being used as the primary one. Results for the main outcome were analyzed at 12 months but were recorded until a maximum of 30 months (July 29, 2021), when the trial ended.

### Sample Size Calculation

The sample size was calculated using the Sealed Envelope platform,^39^ considering a success rate of 88% in the control group receiving standard CR. Thus, if there were a true difference of 7% in favor of the experimental arm with hybrid CR, 84 patients per arm were required for us to be 80% sure that the upper limit of a 1-sided 97.5% CI excluded a difference of more than 5% in favor of the standard group. The noninferiority margin of 5% was set at 62% of the difference between the standard or current intervention and usual care (8%), obtained from a systematic review by Anderson et al^40^ balancing study feasibility with an acceptable loss of therapeutic efficacy.

### Statistical Analysis

To test the primary hypothesis, differences in proportions of recurrent cardiovascular events were estimated as absolute risk difference (ARD) with 5% of the noninferiority margin. Intention-to-treat (ITT) and per-protocol (PP) analyses were performed; noninferiority was established if both ITT and PP analyses supported it.^41^ For the PP analysis, 4 levels of adherence to the intervention were compared: 80%, 60%, 40%, and 20% of attendance at supervised CR sessions. The two 1-sided test procedure (TOST) was used to test the noninferiority of hybrid CR compared with standard CR.^41^ Additionally, relative risk (RR) was calculated, and survival analysis was performed to estimate the hazard ratio (HR).

To test the secondary outcomes, both the ARD and RR were estimated for binary outcomes, and mean differences were calculated for continuous outcomes. Data were analyzed using Stata, version 18.0 (StataCorp LLC). For secondary outcomes, significance was set at 2-sided *P* < .05.

## Results

**Figure 1.**
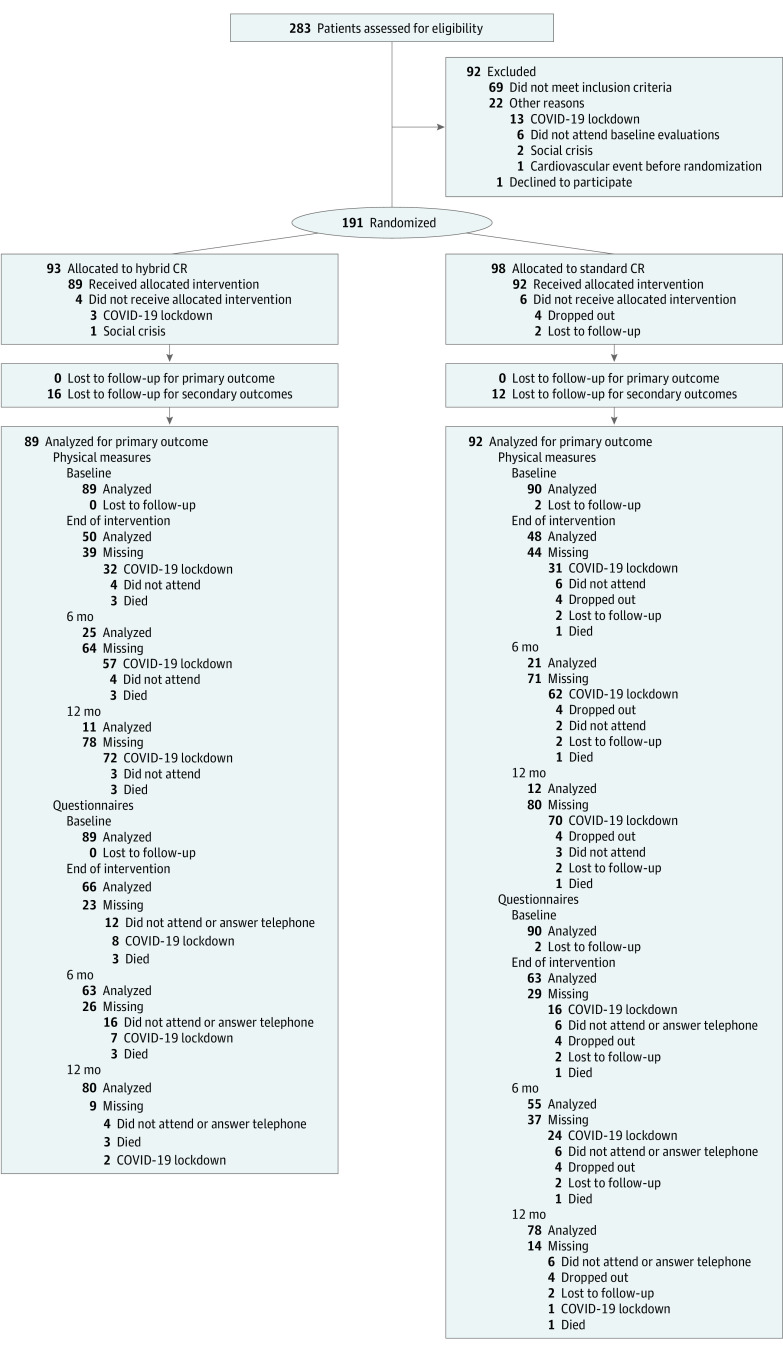
CONSORT Flowchart With CONSERVE Considerations CR indicates cardiac rehabilitation.

A total of 191 participants were included from 283 potential participants, with a mean (SD) age of 58.74 (9.80) years; 46 (24.08%) were female, and 145 (75.92%) were male. These 191 participants were randomly assigned to hybrid CR (n = 93) or standard CR (n = 98), with 89 (95.70%) and 92 (93.88%) in each respective group actually receiving the intervention. In Table 1, the baseline characteristics of the participants by arm are shown, with no important differences between groups, and Figure 1 shows the CONSORT flowchart detailing the reasons for not receiving the assigned intervention and loss to follow-up, with details of the data available for analysis at each follow-up point by outcome group in accordance with the CONSERVE guidelines.^24^ Reasons for the loss of data for physical measurements or questionnaires are reported separately, since from the beginning of the COVID-19 pandemic, the participants could not be on site for the physical measurements but it was possible to administer the questionnaires by telephone when participants agreed.

**Table 1.** Baseline Characteristics by Arm in the HYCARET

Characteristic	Participants^a^		
	All (N = 191)	Hybrid CR (n = 93)	Standard CR (n = 98)
**Demographic**			
Age, mean (SD)	58.74 (9.80)	58.83 (9.54)	58.65 (10.10)
Sex			
Female	46 (24.08)	22 (23.66)	24 (23.71)
Male	145 (75.92)	71 (76.34)	74 (76.32)
Marital status			
Married or unmarried partner	124 (64.40)	61 (65.59)	63 (64.29)
Single	32 (16.75)	12 (12.90)	20 (20.40)
Divorced, separated, or widowed	35 (18.32)	20 (21.51)	15 (15.30)
Highest level of education completed			
Primary or lower	73 (38.22)	36 (38.71)	37 (37.76)
Secondary	63 (32.98)	29 (31.18)	34 (34.69)
Postsecondary	55 (28.80)	28 (30.11)	27 (27.55)
**Cardiovascular risk factors**			
Tobacco use			
Former	28 (14.66)	13 (13.98)	15 (15.31)
Never	147 (76.96)	70 (75.27)	77 (78.57)
Current	16 (8.38)	10 (10.75)	6 (6.12)
Adherence to PA recommendations	127 (66.84)	66 (70.97)	61 (62.89)
Adherence to diet recommendations	155 (82.45)	79 (84.95)	76 (80.00)
BMI, mean (SD)	28.82 (4.10)	28.93 (4.20)	28.71 (4.10)
Waist circumference, mean (SD), cm	99.54 (9.92)	100.13 (10.02)	98.98 (9.85)
Hypertension	130 (68.42)	65 (69.89)	65 (67.01)
Diabetes	61 (32.11)	28 (30.11)	33 (34.02)
Dyslipidemia	79 (41.58)	41 (44.09)	38 (39.18)
**Other clinical characteristics **			
Diagnosis			
Unstable Angina	12 (6.28)	7 (7.53)	5 (5.10)
AMI	154 (80.63)	71 (76.34)	83 (84.69)
Coronary artery disease	25 (13.09)	15 (16.13)	10 (10.20)
Time between event or diagnosis and baseline assessment, mean (SD), d	49.33 (51.82)	49.26 (51.88)	49.5 (52.03)
Treatment^b^			
Only medication	34 (17.89)	18 (19.35)	16 (16.49)
Thrombolysis	10 (5.26)	5 (5.38)	5 (5.15)
Angioplasty	132 (69.47)	63 (67.74)	69 (71.13)
Revascularization surgery	54 (28.42)	29 (31.18)	25 (25.77)
AACVPR cardiovascular risk^c^			
Low	95 (63.33)	45 (61.64)	50 (64.94)
Moderate	33 (22.00)	15 (20.55)	18 (23.38)
High	22 (14.67)	13 (17.81)	9 (11.69)

Abbreviations: AACVPR, American Association of Cardiovascular and Pulmonary Rehabilitation; AMI, acute myocardial infarction; BMI, body mass index (calculated as weight in kilograms divided by height in meters squared); CR, cardiac rehabilitation; HYCARET, Hybrid Cardiac Rehabilitation Trial; PA, physical activity.

^a^ Data are presented as number (percentage) of patients unless otherwise indicated.

^b^ Participants had more than 1 treatment.

^c^ For 40 participants, not all data were obtained to estimate cardiovascular risk.

Results for each outcome of interest at each follow-up point in both arms are displayed in eTable 2 in Supplement 2. By the end of follow-up, 14 participants (7.33%) had experienced a cardiovascular event: 5 in the hybrid CR group (5.38%) and 9 in the standard CR group (9.18%). Specifically, 4 of the 5 participants with a cardiovascular event in the hybrid CR group (80.00%) had a myocardial infarction, of which 2 (50.00%) were fatal and 2 (50.00%) required hospitalization; the fifth participant (20.00%) was diagnosed with heart failure. Of the 9 participants who had an event in the standard CR group, 4 (44.44%) experienced myocardial infarction requiring hospitalization, another 4 (44.44%) experienced heart failure (2 [50.00%] on an outpatient basis and the other 2 [50.00%] due to decompensation requiring hospitalization), and the ninth participant (11.11%) died of pulmonary-related sepsis.

In terms of adherence to supervised CR sessions, the hybrid CR group had 79.14% adherence (736 of 930 supervised CR sessions), which was significantly higher than in the standard CR group, which had 61.46% adherence (1201 of 1954 sessions) (*P* < .001). In the standard CR group, of the 9 participants with reported cardiovascular events, only 1 (11.11%) adhered to more than or equal to 80% of the supervised exercise program, 1 (11.11%) adhered to 60%, 2 (22.22%) adhered to 45%, 1 (11.11%) adhered to 30%, and 4 (44.44%) adhered to less than 20%. In contrast, in the hybrid CR group, of the 5 participants who reported cardiovascular events, 4 (80.00%) adhered to 100% of the supervised exercise program and 1 (20.00%) adhered to 40%.

**Figure 2.**
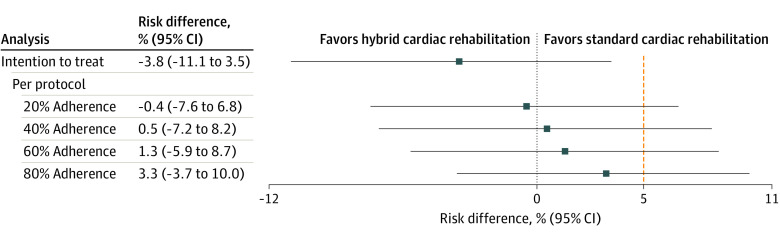
Absolute Risk Differences for the Main Outcome of Recurrent Cardiovascular Events by Intention-to-Treat and Per-Protocol Analyses The no difference threshold corresponds to 0% and the noninferiority limit (orange dashed line) to 5%.

For the main outcome of recurrent cardiovascular events, noninferiority was detected in the ITT analysis, where the upper limit of the 95% CI was 3.52%, not exceeding the noninferiority limit of 5%. This was not the case in the PP analysis, in which the greater the adherence to the intervention, the further away from the no-effect threshold the point estimate of the ARD was found and the further away from the noninferiority threshold of the upper limit of the 95% CI was found (eg, 20% adherence: ARD, −0.35% [95% CI, −7.56% to 6.85%]; 80% adherence: ARD, 3.30% [95% CI, −3.70% to 10.31%]). Figure 2 shows the findings derived from the TOST procedure for both ITT and PP analyses at different levels of adherence to attending supervised CR sessions. Participants in the hybrid CR group had 3.80% (95% CI, –11.13% to 3.52%) fewer cardiovascular events than participants in the standard CR group, and relative risk was 0.59 (95% CI, 0.20-1.68) for the primary outcome. In the worst scenario (upper limit of the 95% CI), participants receiving hybrid CR had a 3.52% higher likelihood of experiencing an event, but this was lower than the 5% established as the noninferiority threshold, indicating that hybrid CR was not inferior to standard CR according to the ITT analysis. The PP analysis showed that all point estimates did not exceed the noninferiority threshold but the upper limits of the 95% CIs exceeded the threshold, indicating that in some patients, the hybrid CR was inferior to the standard CR. Finally, the HR for the ITT analysis was 0.67 (95% CI, 0.22-2.06), with censoring of 5.38% for the hybrid CR arm vs 9.18% for the standard CR arm (*P* = .31).

Table 2 shows the findings derived from the ITT analysis and the PP analysis at 80% adherence to the intervention for all outcomes at each follow-up point. eTables 3 and 4 in Supplement 2 show the results for the PP analyses at the 60%, 40%, and 20% adherence levels.

**Table 2.** Between-Group Effects by Assessment Point in ITT and PP Analyses at Adherence of 80% or Greater to Supervised Sessions

Outcome	ITT analysis^a^			PP analysis at ≥80% adherence to supervised sessions^b^		
	End of intervention	6 mo	12 mo	End of intervention	6 mo	12 mo
**ARD (95% CI)**						
Recurrent cardiovascular events	NAT	NAT	−0.04 (−0.11 to 0.04)	NAT	NAT	0.03 (−0.04 to 0.10)
Adherence to physical activity recommendations	0.07 (−0.03 to 0.17)	0.11 (0.01 to 0.22)	0.04 (−0.06 to 0.14)	0.05 (−0.06 to 0.16)	0.02 (−0.09 to 0.13)	0.01 (−0.13 to 0.11)
Adherence to diet recommendations	0.04 (−0.14 to 0.06)	0.08 (−0.18 to 0.02)	0.02 (−0.11 to 0.08)	0.01 (−0.10 to 0.12)	0.09 (−0.22 to 0.04)	0.05 (−0.16 to 0.07)
**RR (95% CI)**						
Recurrent cardiovascular events	NAT	NAT	0.59 (0.20 to 1.68)	NAT	NAT	2.42 (0.28 to 20.97)
Adherence to physical activity recommendations	1.07 (0.96 to 1.20)	1.14 (1.01 to 1.30)	1.05 (0.94 to 1.17)	1.05 (0.93 to 1.19)	1.02 (0.90 to 1.16)	0.98 (0.87 to 1.11)
Adherence to diet recommendations	0.67 (0.22 to 2.00)	0.37 (0.10 to 1.35)	0.85 (0.35 to 2.10)	1.19 (0.30 to 4.70)	0.30 (0.06 to 1.56)	0.58 (0.15 to 2.18)
**Difference, mean (95% CI)**						
Heart QOL						
Global	−0.06 (−0.22 to 0.11)	0.06 (−0.09 to 0.20)	−0.04 (−0.22 to 0.13)	0.01 (−0.20 to 0.22)	0.09 (−0.09 to 0.27)	0.06 (−0.13 to 0.24)
Physical function	−0.07 (−0.22 to 0.08)	−0.00 (−0.16 to 0.16)	−0.11 (−0.29 to 0.60)	−0.04 (−0.23 to 0.16)	−0.00 (−0.20 to 0.19)	−0.07 (−0.27 to 0.13)
Emotional function	−0.05 (−0.24 to 0.14)	0.09 (−0.07 to 0.25)	−0.01 (−0.20 to 0.19)	0.04 (−0.20 to 0.28)	0.14 (−0.05 to 0.33)	0.12 (−0.08 to 0.34)
EQ-5D, VAS	−2.22 (−8.12 to 3.69)	−0.45 (−7.76 to 6.86)	−0.08 (−5.79 to 5.63)	ISS	1.26 (−0.98 to 2.31)	−0.84 (−1.22 to 1.01)
Exercise capacity, m	26.12 (−26.34 to 78.59)	40.58 (−13.63 to 94.78)	ISS	−16.77 (−74.37 to 40.83)	ISS	ISS
Muscle strength, kg	−1.02 (−2.95 to 0.91)	1.07 (−2.19 to 4.33)	ISS	ISS	ISS	ISS
Return to work	NAT	NAT	5.25 (−45.48 to 55.99)	NAT	NAT	NAT

Abbreviations: ARD, absolute risk difference; CR, cardiac rehabilitation; EQ-5D, EuroQol 5-dimension measure; ISS, insufficient sample size; ITT, intention-to-treat; NAT, not assessed at this time point; PP, per-protocol; QOL quality of life; RR, relative risk; VAS, visual analog scale.

^a^ The hybrid CR arm included 93 participants, and the standard CR arm, 98 participants.

^b^ The hybrid CR arm included 71 participants, and the standard CR arm, 43 participants.

Finally, 3 participants in the hybrid CR group (3.22%) reported exercise-related adverse events (1 [33.33%], angina without need for emergency care; 2 [66.67%], nonspecific symptoms). Thirteen participants in the standard CR group (13.27%) experienced adverse events (5 [38.46%], dizziness; 2 [15.38%], substantial palpitations; 1 [7.69%], syncope; 1 [7.69%], hypertensive crisis; 1 [7.69%], hypotension; 1 [7.69%], angina without the need for emergency care; 2 [15.38%], nonspecific symptoms).

## Discussion

This randomized clinical trial tested a CR model incorporating low-tech delivery modes, which was well suited for infection prevention and control, consistent with models widely implemented worldwide during the pandemic.^42^ However, this trial, like many others, was hampered by COVID-19. The findings indicate that hybrid CR was noninferior to standard CR in ITT analysis but not in PP analysis. While it is suggested that noninferiority should be demonstrated in both analyses, 2 important considerations arose. First, due to the COVID-19 pandemic, recruitment of new participants was suspended, leading to a revised sample size based on different assumptions and potentially affecting statistical power. Second, PP analyses exclude participants who do not adhere to treatment; in our trial, adherence differed significantly by trial arm, raising the possibility of exclusion bias.^41^ As previously stated, in the standard CR group, of the participants with reported cardiovascular events, only 1 adhered to more than or equal to 80% of the supervised exercise program, and of the 5 participants in the hybrid CR group who reported cardiovascular events, 4 adhered to 100% of the supervised exercise program. This diluted the event differences in favor of the hybrid CR group, as suggested by the ITT analysis.

Other trials of hybrid CR are limited, particularly in low-income settings for patients with coronary conditions. Additionally, interventions vary widely and often cannot be directly compared with our study’s approach given the inherently complex nature of CR.^43^ One of the first such studies in the US examined hybrid CR in 80 patients with low to moderate cardiac risk.^44^ The study’s intervention, similar to ours but extended by 3 months (totaling 6 months), found that the low-cost hybrid model was as effective as a traditional CR protocol in physiological outcomes, exercise adherence, and program participation.

Two recent studies^45,46^ also found similar conclusions regarding comparable effects including feasibility, safety, and effectiveness outcomes, such as quality of life, mental health, and functional capacity. Pakrad et al,^45^ in a study involving 107 patients with coronary artery bypass graft in Iran, compared a 12-session supervised CR program over 1 month with the same program supplemented by 3 months of smartphone-delivered care involving 24 contacts through a mobile app. While the intervention was hybrid and implemented in a middle-income setting, it had a longer duration compared with the control group. A strength of HYCARET is that although we integrated technology in a more basic yet arguably feasible and adoptable manner through telephone calls and text messages, we also ensured an equivalent 3-month follow-up period for both groups to eliminate alternative explanations for our findings. A study by Meslet et al^46^ in France involved 60 patients recovering from an acute coronary syndrome within the past 3 months. Unlike HYCARET, that study compared a fully center-based CR control group with a partially center-based CR group. The experimental group initially had only 5 sessions of supervised CR followed by the remainder at an equipped sport and community center. In addition, 2 systematic reviews^47,48^ involving other patients showed comparable effectiveness between hybrid and standard CR in terms of functional capacity but contradictory results in relation to quality of life, without considering the occurrence of cardiovascular events as an outcome of interest.

The current trial contributes to the literature in several ways. It was designed with a noninferiority hypothesis, which distinguishes it from the previously mentioned trials,^45,46^ in which a superiority hypothesis was assumed and, in most cases, equivalence conclusions were reached. Also, from a more clinical point of view, fatal and nonfatal cardiovascular events served as the main outcome, and there was a long, 1-year follow-up; these are unique strengths in the cardiac rehabilitation and exercise field. Despite the need for CR being greater and resources being fewer (so that leveraging technology in CR may be more cost-effective) in low-resource settings,^49^ this is 1 of few hybrid CR trials conducted in a low-resource setting; CR interventions designed in higher-resource settings are generally not transferable for multilevel reasons.^50^ In the HYCARET model, the supervised phase minimized resource requirements (eg, professionals and equipment), and the latter, unsupervised phase was mobile telephone supported, which is potentially a more accessible, efficient, and feasible intervention for settings such as in Latin American countries.^50^

### Limitations

This study has limitations, and caution is warranted when interpreting these results. First, the adjustment to sample size and the substantial loss to follow-up for secondary outcome measures due to extenuating circumstances limited power and precision in effect estimates. Second, the feasibility rationale for setting a noninferiority limit may be questionable, but setting a limit considering all clinical factors and patient perspectives would make this and almost all noninferiority trials unfeasible.^51^ These 2 limitations may be overcome as more interventions like this one are studied and results are aggregated in future meta-analyses.^51^ Third, while the trial was conducted in a Latin American country, generalizability to other low-resource settings and other regions of the world cannot be known. Fourth, the fact that there was no concordance between the findings of the ITT and PP analysis weakens confidence in the noninferiority findings despite the aforementioned justification about the participants eliminated in the PP analysis.

## Conclusions

The results of this trial showed that a hybrid CR model that aims to enable patients to achieve cardiovascular disease self-management, first through a supervised, center-based phase and then through a second phase of follow-up via mobile telephone, may be noninferior to a standard, center-based CR model, primarily in terms of the recurrence of cardiovascular events and potentially in terms of intermediate outcomes such as HRQOL, functional capacity, cardiovascular risk factors, muscle strength, heart-healthy behavior, return to work, and exercise-related adverse events. In terms of adherence to supervised sessions, the hybrid CR model may be superior to the standard model of CR. With regard to broad implications, considering the currently available evidence as well as the findings of the current study, where resources permit and where supervised CR and unsupervised CR are reimbursed,^42^ clinical factors and patient preferences should be considered when determining the best model of CR for patients, as supervised and hybrid CR are likely also equivalent in terms of patient outcomes in low-resource settings. Moreover, the lower cost of hybrid CR may enable greater capacity to treat the many patients in need in these settings.

## Notes

Supplement 1.
Trial Protocol

Supplement 2. **eMethods 1.** Inclusion and Exclusion Criteria **eTable 1.** Study Centers, Location, Ethics Committee Approvals, and Amendments **eMethods 2.** Text Message and Phone Call Content in HYCARET **eTable 2.** Measurements for Each Outcome at Each Follow-Up Point According to the Assigned Group **eTable 3.** Between-Group Effects by Assessment Point in PP Analysis by the Level of Adherence (60%, 40%, 20%) to the Intervention (Binary Outcomes) **eTable 4.** Between-Group Effects by Assessment Point in PP Analysis by the Level of Adherence (60%, 40%, 20%) to the Intervention (Continuous Outcomes)
eReference

Supplement 3.
HYCARET Investigators

Supplement 4.
Data Sharing Statement
