# Supplementary material for: Hybrid Cardiac Rehabilitation Program in a Low-Resource Setting: A Randomized Clinical Trial
Source: JAMA Netw Open. 2024 Jan 9;7(1):e2350301. doi: 10.1001/jamanetworkopen.2023.50301 (PMC10777264; doi:10.1001/jamanetworkopen.2023.50301)
Supplement: Supplement 1. — Trial Protocol [file jamanetwopen-e2350301-s001.pdf]

Effectiveness of a hybrid cardiac rehabilitation model for coronary artery disease patients in a low-resource setting. HYCARET randomized clinical trial.

Original Protocol. HYCARET Study

FONDECYT Nº 1181734 • Contacto: hycaret@ufrontera.cl

PROPOSAL ABSTRACT:

**Name of Principal Investigator:**	**Pamela Serón Silva**
**Proposal Title:**	**Effectiveness of a hybrid exercise-based cardiac rehabilitation program. Randomized, multicenter, and non-inferiority clinical trial.**

Background: Cardiac rehabilitation programs are designed as a secondary prevention intervention in patients surviving cardiovascular events. These programs are widely established, and their effectiveness and cost-effectiveness proven, given they reduce cardiovascular mortality and subsequent hospitalizations. In spite of this, this intervention remains underutilized, not only because of low patient referral, but also because of the scarcity of existing programs (especially in middle and low resource settings such as the Latin American context). As cardiovascular disease is the condition that produces the highest mortality and disability-adjusted life-years in the world, with major subsequent impact on the countries' economies, there is an urgent need to create more accessible delivery models of cardiac rehabilitation programs in order to increase the efficiency of health systems and improve population health.

Hypothesis: A hybrid exercise-based cardiac rehabilitation model, which include 10 face-to-face sessions plus distance monitoring through text messages and phone calls is not inferior in effectiveness to the standard model.

General goal: To evaluate if an abbreviated exercise-based cardiac rehabilitation program is not inferior to the standard exercise-based cardiac rehabilitation program.

Specific goals:

Primary goal: To evaluate if the prevention of recurrent cardiovascular events is not inferior in the hybrid exercise-based cardiac rehabilitation programs in comparison to the standard program.

Secondary goals:

- To compare the effectiveness of the abbreviated cardiac rehabilitation programs with the standard program in terms of:
    - cardiovascular risk factor control (dyslipidemia, hyperglycemia, hypertension, obesity)
    - exercise capacity
    - adherence to physical activity recommendations
    - adherence to diet recommendations
    - health-related quality of life
    - exercise related adverse events

Methodology: A non-inferiority, pragmatic, multicenter, parallel, single blinded, randomized clinical trial will be conducted. 314 patients with coronary artery disease including Acute Coronary Syndrome (Unstable Angina, Myocardial infarction with or without ST elevation) or stable coronary vessel disease diagnosed by coronariography or stress test, who have received medical treatment, thrombolysis, angioplasty or revascularization surgery will be recruited consecutively, with written informed consent. Participants will be assigned to experimental or standard rehabilitation programs using a permuted blocked randomization scheme. Concealment of assignment will be preserved. The experimental cardiac rehabilitation program includes medical evaluations, 10 face-to-face exercise sessions, diet and smoking counseling, and distance monitoring with text messages and phone calls, delivered by a physiotherapist. The standard cardiac rehabilitation consists of medical evaluation, 20-24 face-to-face exercise sessions, as well as education sessions about diet and smoking, delivered by a nurse, nutritionist and physiotherapist. The main outcome is a composite of cardiovascular mortality and hospitalizations due to cardiovascular causes (non-fatal stroke, non-fatal myocardial infarction, heart failure, and need of revascularization surgery). Secondary outcomes are cardiovascular risk factor control, exercise capacity, adherence to physical activity and diet recommendations, health-related quality of life and exercise-related adverse events. The outcomes will be measured at the end of intervention, at 6 months, and at 12 month follow-up from recruitment. Only main outcome will be a maximum of three year of follow-up. The sample size was calculated considering 5% of non-inferiority limit. Per protocol and intention to treat analysis will be considered. A survival analysis will be run for the primary outcome. One interim analysis is planned when 50% of patients have completed the evaluation at the end of the first year of follow-up.

Expected results: We anticipate that if non-inferiority is demonstrated, a more suitable and affordable model of cardiac rehabilitation will be available for patients and the health system, to increase coverage, save resources, and improve cardiovascular health indicators.

PROPOSED RESEARCH

1. Background.

1.1. Cardiovascular disease. Epidemiology and disease burden.

Cardiovascular disease (CVD) is the leading cause of morbidity and mortality in the world, of which ischemic cardiomyopathy produces the highest mortality and disability-adjusted life-years (1). In the Americas, CVD contributes to 33.7% of total deaths (2) and it is estimated that the number of deaths in the region attributable to CVD will increase by more than 60% from the year 2000 to 2020, compared with an increase of only 5% in the developed world (3). Specifically in Latin America and the Caribbean, ischemic heart disease is the predominant form of CVD, with an adjusted mortality of 66.4 per 100,000 persons (4). In Chile, CVD accounts for 66.7% of diseases, making it the second most common condition, for which the most life-years are lost due to premature death (5).

1.2. Secondary Prevention. Purpose, elements and goals achievement.

Once CVD has been established, the fundamental objective is to prevent mortality, recurrent cardiovascular events, and other complications through secondary prevention programs that include drug therapy, healthy lifestyle education, and control of risk factors such as high blood pressure, cholesterol, as well as diabetes mellitus(6,7). While these recommendations are widely known, their achievement is not as expected, as evidenced by the Prospective Urban Rural Epidemiology (PURE) study which reported that only 35.1% (95%CI, 29.6% - 41.0%) of patients maintain high levels of physical activity; 39.0% (95%CI, 30.0% - 48.7%) follow a healthy diet; and 18.5% (95%CI 17.6% - 19.4%) continue smoking despite a cardiovascular event. The achievement of these heart-health goals is even lower in low-income countries (8). This scenario is reproduced in the South American region and in Chile, where it has been reported that 50% of patients suffering a myocardial infarct achieve 4 of 8 established goals, the worst being having a normal body mass index (BMI) (22%) and performing physical activity (23.9%) (9).
To achieve the secondary prevention goals, the interventions currently in use need to be implemented through formal programs that promote healthy lifestyle as well as adherence to medical recommendations and drug therapy. One intervention that is recommended by international guidelines is Cardiac Rehabilitation (10,11), which is an efficient, comprehensive way to deliver secondary prevention and promote better outcomes.

1.3. Cardiac Rehabilitation. Effectiveness, cost-effectiveness, availability, and barriers.

Cardiac rehabilitation programs include medical evaluation, education, counselling, and prescription of exercise as their key components (12). Guidelines for cardiac rehabilitation strongly recommend supervised exercise / individually-prescribed physical activity, based on high-level evidence which support effectiveness. The exercise programs in these clinical guidelines including similar characteristics: long programs of at least two or three months duration, with two to five exercise sessions per week, consisting of low to moderate intensity aerobic training combined with resistance exercises in sessions between 30 to 60 minutes (13).
The most rigorous systematic review that evaluates cardiac rehabilitation, which included 63 clinical trials comprised of 14,486 randomized patients, demonstrates that it reduces cardiovascular mortality (RR=0,74; CI95% 0,64 to 0,86) and recurrent hospitalization (RR=0,82; CI95% 0,70 to 0,96), and improves quality of life. The benefits in outcomes were independent of patients' specific diagnosis, type of cardiac rehabilitation (exercise only vs comprehensive rehabilitation) dose of exercise, length of follow-up, trial publication date, setting (center vs home-based), study location (continent), sample size and risk of bias (14).

Despite this knowledge, there are barriers to delivery of exercise-based cardiac rehabilitation programs. Indeed, cardiac rehabilitation is under-utilized (including in Chile) when compared with other recommended secondary prevention interventions, such as medication. The barriers to delivery of cardiac rehabilitation programs include those that are patient-oriented (e.g., patient refusal), provider-oriented (e.g., provider fails to refer patient to cardiac rehabilitation), and others that are related to the health care system (e.g., lack of a cardiac rehabilitation programs, lack of insurance coverage, etc.) (12). Indeed worldwide, only 38.8% of countries have cardiac rehabilitation programs. Specifically, 68% of high-income and 23% of low/middle-income countries offer cardiac rehabilitation programs to patients with CVD (15). In Chile, the

existing cardiac rehabilitation programs cover only 5% of patients who need them, and these programs are mainly in the private system and in the country's capital (16). This is a very similar situation to that of other Latin American countries (17), revealing major inequalities of access.
Since one of the barriers to delivery is the cost of implementation of cardiac rehabilitation programs, economic evaluations of cardiac rehabilitation have been conducted, across many different settings. A systematic review that included 22 such economic evaluations supported the implementation of this service (18). Another review, which includes studies from low- and middle-income countries, shows only two cost-effectiveness evaluations in Latin America, only in patients with heart failure. These reports from Brazil (19) and Colombia (20) show that cardiac rehabilitation is highly cost-effective, but the review authors are emphatic in concluding that given the countries' limited budget, affordable models need to be designed, especially for countries or settings with low income levels (21).

Specifically in Chile, our group conducted a cost-utility analysis which concluded that, considering the cost-effectiveness threshold of 1 Gross Domestic Product per capita, exercise-based cardiac rehabilitation is highly cost-effective for the public health system, and should be the standard of care cardiovascular secondary prevention. In this study, a standard model of exercise-based cardiac rehabilitation (as evaluated in the Cochrane Systematic Review by Anderson) resulted in an incremental cost-effectiveness ratio (ICER) of US$ 722.34 (CL$ 480,359.66) per quality-adjusted life year (QALY), compared with that of standard care (FONIS SA14ID0005).

One aspect that is necessary to consider, which we grappled with in our economic evaluation, is that one element that introduce structural uncertainty in the modelling process is the configuration of the cardiac rehabilitation service; the costs differ in different regions, countries and settings, and the effectiveness could vary as well. Indeed, the variability in the cost of cardiac rehabilitation delivery is wide around the world. According to a recent review, costs ranged from US$294 in the United Kingdom to US$12,409 in Italy in high-income countries, and from US$146 in Venezuela to US$1,095 in Brazil in middle-income countries. These differences were explained principally by differences related to facilities, personnel involved, and session dose (all values in this review are standardized according to purchasing power parity) (22). In our economic evaluation, we valued standard exercise-based cardiac rehabilitation in Chile at US$102.07, this did not consider pharmacologic therapy (because it is considered usual care), which would increase the cost near to US$1,500.

1.4. Cardiac Rehabilitation Models.

Several approaches to lowering the cost and increasing accessibility of cardiac rehabilitation have been described, such as delivering the interventions in unsupervised settings, using information and communication technology, task-shifting to lower-cost healthcare personnel, and offering a lower dose (i.e., fewer sessions). Other strategies include using lower-cost exercise equipment, and ceasing ECG-monitoring during exercise sessions (22,23). The way that these strategies are implemented in the programs formulates several alternative models to the traditional cardiac rehabilitation.

A systematic review characterized these models, and evaluated their effectiveness against traditional supervised programs. From 8 broad categories of alternative models identified, two were shown be effective. First, the multifactorial individualized telehealth model produced similar reductions in cardiovascular risk factors compared with traditional programs; however there is a lack of cost-effectiveness data and information about harder outcomes such as re-hospitalizations and subsequent cardiac events. Second, the community or home-based cardiac rehabilitation models were shown to result in minimal differences with traditional programs in terms of mortality and cardiovascular event rates (24).

One strategy increasingly proposed and used is delivery of cardiac rehabilitation via the internet. Considering its newness however, availability and quality of evidence demonstrating effectiveness is low when compared with the high-quality evidence that support the effectiveness of traditional programs, or even through personal contact via telephone using coaching (24,25).

Besides this several settings approaches, and considering resources and task-shifting, countries such as the Netherlands and United Kingdom, recommend that a general physician, exercise physiologist, physiologist, physiotherapist, allied healthcare provider, or another staff member trained in exercise prescription would be sufficient to supervise exercise sessions (26,27).

49
50

The most recently-proposed model is that designed by the International Council of Cardiovascular Prevention and Rehabilitation (ICCPR) entitled Cardiac Rehabilitation Delivery Model for Low-Resource Settings. Therein, strategies to deliver all core components of cardiac rehabilitation, but adapted to low-resource settings are recommended. In this proposal there is an algorithm for cardiac rehabilitation delivery based primarily on availability of healthcare personnel. In the second scenario of this algorithm, how each component of cardiac rehabilitation (e.g., assessment, lifestyle management, medical management, and structured exercise) can be delivered by an allied healthcare worker (e.g., physiotherapist) is outlined, and the approaches proposed are feasible to implement in a non-hospital environment. Additionally, this consensus statement offers direction for adaptation according to setting, including mobile technology (23).
In our economic evaluation, we generated a third model in order to evaluate the cost-utility of this Model for Low-Resource Settings. The value of one entire program for one patient resulted in US$46.16, which is 45% cheaper than the Standard Model; however effectiveness data is not yet available considering the consensus is quite recent, thus we had to run probabilistic analysis using the effect reported for the Standard Model resulting in considerable uncertainty in our conclusions.

51

1.5. Summary of rationale and knowledge gap.

Considering the current scenario described previously, where:

- There is an important disease burden caused by the growing epidemic of CVD.
- That from the point of view of the nation of Chile, it is necessary improve cardiovascular health in order to mitigate the epidemic.
- That from point of view of patients suffering a cardiovascular event, they need to prevent recurrent events, complications, death, and enhance their quality of life.
- Cardiovascular secondary prevention indicators have been poorly achieved.
- There are established interventions that can improve outcomes. Specifically cardiac rehabilitation is a strategy to organize secondary prevention, promoting medication adherence and healthy lifestyles through core components, with the exercise as the fundamental intervention.
- The standard model of exercise-based cardiac rehabilitation has been proven as an effective intervention to prevent cardiovascular death and hospitalizations, but most of this evidence stems from other regions than Latin America, and high-incomes countries in particular (14).
- The exercise-based cardiac rehabilitation programs are under-utilized and one of the barriers to deliver the service is cost involved and capacity (15).
- There are several alternative models of cardiac rehabilitation which may mitigate these barriers, which have been evaluated, but there remains gaps in evidence supporting some (24).
- That from point of view of the health systems, and in a resource-constrained era, we need to increase coverage to patients while at the same time that not increasing expenditures, such that the system is more efficient with greater impact at the population-level (22).
- A Low-Resource Setting Model of cardiac rehabilitation has been designed by an international council. This model recommends delivery of all core components adapted according to the available resources, in alternative settings and by lower-cost personnel (28).
- In spite of the Low-Resource Setting Model has been designed following a rigorous methodology in order to establish recommendations of the minimums, the fact that consider less supervised exercise sessions, less equipment, less monitoring, less education sessions, and less professional availability, could be make it less effective than standard model which has been evaluated against usual care. It needs to be tested in a real-world setting.

52
53

Is necessary to evaluate the effectiveness of a model of exercise-based cardiac rehabilitation more accessible, cheaper, more efficient, and designed for our local settings.
As the current knowledge is that the Standard Model of exercise-based cardiac rehabilitation is effective in reduce cardiovascular mortality and hospitalizations, compare the new model against usual care is not ethically possible, and to conduct a non-inferiority clinical trial seems to be the best alternative to generate local evidence, with better quality and minimal bias.
We anticipate that if non-inferiority of a lower-resource model is demonstrated, a more suitable and affordable model of cardiac rehabilitation will be available for patient and the health system in order to increase coverage, save resources, and improve cardiovascular health outcomes.

54
55
56

2. **Hypothesis:** A hybrid exercise-based cardiac rehabilitation model, which include 10 face-to-face sessions plus distance monitoring through text messages and phone calls is not inferior in effectiveness to the standard model. In other words, a program with less supervised sessions, using fewer human resources, and less equipment, but complemented with information and communication technology will not result in more recurrent cardiovascular events (is not inferior) than the standard model of cardiac rehabilitation.

3. Research goals:

3.1 General goal: To evaluate if a hybrid exercise-based cardiac rehabilitation program is not inferior to the standard exercise-based cardiac rehabilitation program.

3.2. Specific goals:

3.2.1. Primary goal:

To evaluate if the prevention of recurrent cardiovascular events is not inferior in the hybrid exercise-based cardiac rehabilitation programs in comparison to the standard program.

3.2.2. Secondary goals:

- To compare the effectiveness of the hybrid cardiac rehabilitation programs with the standard program in terms of:
    - cardiovascular risk factor control (dyslipidemia, hyperglycemia, hypertension, obesity)
    - exercise capacity
    - adherence to physical activity recommendations
    - adherence to diet recommendations
    - health-related quality of life
    - exercise-related adverse events

4. Methodology.

4.1 Design.

A non-inferiority, pragmatic, multicenter, 2 parallel arm, single-blinded, randomized clinical trial will be conducted.

In this non-inferiority study, the objective is to demonstrate that a hybrid exercise-based cardiac rehabilitation model is not inferior (i.e., equivalent or possibly superior) than a standard model. This trial will be pragmatic because it examines the outcomes of the experimental intervention compared with a standard intervention under circumstances which closely approximate the real world. Patients will be recruited in 6 health centers. Personnel undertaking outcome assessment will be blinded to group allocation. Assessment points are shown in the Figure. Finally, randomization process will allow to balance in both groups all, known and unknown, demographic and clinical characteristics that could confuse the association between intervention and results. The trial protocol will be registered (www.clinicaltrial.gov), and reported in accordance with CONSORT (29) including the extensions for non-pharmacologic treatment interventions, non-inferiority and equivalence, and pragmatic trials.

4.2. Study population.

The reference population is coronary disease patients. The accessible population is patients that attend one the 6 health centers involved in the study: Complejo Hospitalario San José, Hospital San Juan de Dios, Hospital Padre Hurtado, Hospital Clínico de la Universidad de Chile, Hospital Clínico San Borja Arriarán y Hospital Dr. Hernán Henriquez Aravena. The sample will consist of patients in the above centers that meet the following eligibility criteria:

64

Inclusion criteria:

- Subjects 18 year old or over.
- Patients with coronary artery disease, including Acute Coronary Syndrome (Unstable Angina, Myocardial infarction with or without ST elevation) or stable coronary vessel disease diagnosed by coronariography or a stress test.
- Patients treated medically (i.e., medication only) or by thrombolysis, angioplasty, or revascularization surgery.
- Patients able to attend the health center almost twice a week over three months.
- Patients who owns a mobile phone.
- Patients that consent to participate in the study through signing an informed consent form.

Exclusion criteria:

- Patient has a planned repeat cardiac or other procedure in next 12 months.
- Explicit contraindication to perform exercise based on American College of Sport Medicine (30).
- Patients with comorbidities such as dementia, blindness, deafness, serious mental illness, and frailty.
- Musculoskeletal disease that precludes the patient from performing exercise.

4.3. Sample size.

If there is a true difference in favour of the hybrid exercise-based cardiac rehabilitation of 7% in hospitalization rate, then 254 patients are required (127 per group) to be 80% sure that the upper limit of a one-sided 97.5% confidence interval will exclude a difference in favour of the standard exercise-based cardiac rehabilitation group of more than 6%. Considering anticipated loss of follow-up (10%) and the inclusion of an interim analysis that penalizes the power (interim analysis adjustment using Pocock boundary), 314 patients will be recruited (157 per group).

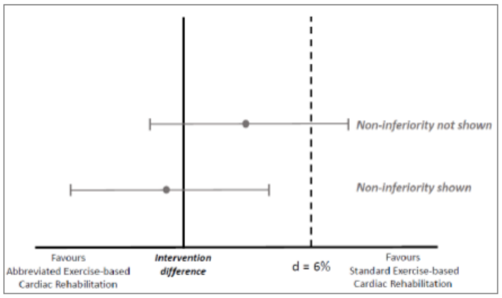

This sample size considered the hospitalization and cardiovascular mortality outcomes that was reported in the systematic review by Anderson in 2016 (14), which totaled 18% in the first year of follow-up in those randomized to exercise-based cardiac rehabilitation. The non-inferiority margin was stablished as is suggested by the Food and Drugs Administration (FDA) (31), which is 50% of the difference between the standard or current therapy (i.e., standard cardiac rehabilitation) and the placebo (or usual care) obtained from a meta-analysis, in this case the same by Anderson (12%).

65

66

4.4. Recruitment and randomization

The patients will be invited to participate in this clinical trial in the discharge moment, during the first outpatient visit since discharge or emergency attention, or when a coronariography or a stress test has been performed. All information about the study will be delivered by a nurse or physiotherapist in charge on this task exclusively. When the patients consent to participate in the study an initial evaluation will be performed in order to collect baseline sociodemographic and clinical characteristics. The assigning to the experimental or control group will be by permuted blocked randomization. Concealment of assignment will be preserved by an opaque closed envelope.

4.5. Experimental and comparison groups.

The participants in the experimental group will be randomized to a hybrid cardiac rehabilitation program adapted from the "Cardiac Rehabilitation Delivery Model for Low-Resource Settings" proposed by the International Council of Cardiovascular Prevention and Rehabilitation Consensus Statement (23,28). This program will be delivered by an exercise specialist (physiotherapist). Participation of a nurse, nutritionist, and psychologist is expected in the design phase of counseling materials and as consultants. A referral and consultant physician is considered. Content of the voice calls and text messaging will be extracted from a bank of 137 suggesting (32). The core components in this model are:

1) Initial comprehensive assessment: includes evaluation about physical activity, diet, tobacco consumption, overweight/obesity, CVD knowledge, depression screening, return to work, and medications. Additionally levels of lipids, blood pressure, and glycaemia will be reviewed.

67

68

69

2) Lifestyle risk factor management: one diet and smoking counseling session (as applicable according to initial assessment results) will be provided by the physiotherapist during the second and third session of exercise. If necessary, a referral to mental health department will be made.
3) Exercise sessions: exercise testing with a six-minute walk test (6MWT) will be performed at the beginning of the program to develop an individualized prescription. Then, 10 face-to-face exercise sessions (twice a week) of aerobic and resistance training will ensue, supervised by a physiotherapist. Exercise sessions last 10 minutes at the beginning of the program and are progressed to 60 minutes by the end as tolerated. There will be 5-10 patients per professional in each session. One of the principal purposes of these sessions is to develop patient self-management related to the physical activity habit, and educate them how to monitor exercise intensity at home and in daily life using heart rate and the Borg scale or perceived exertion.
4) Transition to unsupervised phase: after 5 weeks with face-to-face exercise sessions all patients will be monitored through mobile technology. Delivery methods will include voice calls and text messaging once a week through to twelve weeks from program initiation. The content will promote physical activity, healthy diet, and medication adherence.

The participants in the control group will receive the standard cardiac rehabilitation that is delivered in participating centers. These programs accounts with physicians, nurses, nutritionists and physiotherapists. The programs will be standardized in participating centers in accordance with guidelines, and will be consistent with initial comprehensive assessment and lifestyle risk factor management as is described for experimental group. Differentially, this programs provide medical evaluation, education sessions about diet and smoking, and the exercise sessions are delivered in 10-12-weeks program.

4.6. Outcomes, measures and follow-up.

The **primary outcome** is recurrent cardiovascular events during a minimum of 12 month follow-up. This outcome is a composite of cardiovascular mortality (defined as death by stroke, myocardial infarction or heart failure) and hospitalizations due to a cardiovascular cause (non-fatal stroke, non-fatal myocardial infarction, heart failure, and need for revascularization surgery). Death occurrence will be monitored by study personnel through review of a public registry (Servicio de Registro Civil e Identificación of Chile). The death certificate and any associated medical documentation will be copied for consideration by the adjudicating committee. Hospitalization occurrence will be assessed by study personnel through chart review at each participating center and supplemented by phone calls to participants (in case they received care at another center) every two months, using a standardized script. When the participant reports a hospitalization, all associated documents (i.e., tests and exam reports) will be collected from clinical charts in each center.
A central adjudication committee will review all materials. This committee will be composed of three clinician-scientists (at least one will be a cardiology specialist) blinded to participant allocation. Members will make the final decision whether the event is definitive, possible, probable, or if it is rejected, and specify the final hospitalization diagnosis with corresponding ICD-10 code.

Secondary outcomes are:

- Cardiovascular risk factor control: the routine lipids and glycaemia test results will be extracted from charts. Methods are the standards in each center. Blood pressure will be assessed at each visit during the follow-up by trained personnel measuring blood pressure three times at 30-second intervals using the standard digital sphygmomanometer with the corresponding cuff size (33). Weight will be measured with a standing scale supported on a steady surface with participants wearing only underwear. Height will be measured on the Frankfort plane positioned at a 90° angle against a metric tape mounted on a wall. These will be used to compute body mass index, and hence obesity. Waist circumference will be measured at 1 centimeter above the navel at minimal respiration.
- Exercise capacity: the indicators for exercise capacity will be "aerobic capacity" evaluated through the 6MWT and "muscle strength" evaluated through a grip strength protocol. Both protocols will be administered by trained staff. The 6MWT will be performed using a 30 meter internal flat corridor with 2 cones marking the distance limits. Patients will be instructed to walk (no running or jogging) as much as possible for 6 minutes. Rest pauses will be allowed as many times as necessary, but the subject should resume walking as soon as possible. Total distance covered during the test will be recorded. All the procedures will be conducted in accordance with the ATS Statement (34). The grip strength protocol will be executed using a Jamar dynamometer, according to a standardized method. Patients will be instructed to sit in a chair with armrests with the shoulder adducted, elbow articulation

flexed at 90° angle, forearm in neutral position and wrist between 0 and 30° of dorsiflexion. In this position, the subjects will be asked to perform 3 maximal effort trials with each hand; the highest value will be considered (35,36).

- Adherence to physical activity recommendations: The World Health Organization (WHO) recommends that adults (including those with CVD) 18 years old or over perform 150 minutes of moderate-intensity aerobic activity or 75 minutes of vigorous activity per week, or a combination of both. The activity must be practiced in >10 minutes bouts in order to be considered. The International Physical Activity Questionnaire (IPAQ) will be administered by trained personnel, which has been validated and used in the local setting (37–39). This instrument assesses physical activity of moderate and vigorous intensity in activities related to work, domestic labour, active transportation, and leisure time, and hence can quantify the energy spent in MET-minutes/week for categorization in accordance with the WHO recommendation.
- Adherence to diet recommendations: Participant adherence to the Mediterranean diet will be measured by the MEDAS, an adaptation of a previously validated 9-item index (40). The 14-item screener includes 5 additional items that facilitate rigorous assessment of adherence to the traditional Mediterranean diet in the present population. Each question is scored 0 or 1. This questionnaire was developed for the Prevención con Dieta Mediterránea (PREDIMED) study (41). Also, the food-frequency questionnaire (FFQ) will be administered by trained personnel to assess food patterns and habits, which will enable recall of the necessary information to determinate de MEDAS. FFQ is the most common tool for use in epidemiological studies and the clinical setting. In Chile, it was administered in the 2010-2011 Food Consumption National Questionnaire (42), and in two other epidemiological studies conducted by our group where it has been validated (43,44).
- Health-related quality of life: Trained personnel will apply the widely used EuroQol five-dimensional three-level (EQ-5D-3L) instrument. It has already been validated, and the utility values have been established for the Chilean population (45).
- Exercise related adverse events: adverse events during exercise, such as myocardial ischemia or malignant arrhythmias, will be communicated to the monitor of the study. Serious adverse event, as death in the exercise session, will be reported to corresponding ethic committee and monitor. All events will be registered for the interim and final analysis.
- Adherence: attendance at each supervised session, and also adherence to the calls in the intervention group will be collect.

All outcomes will be measured at the beginning of the program, at the end of intervention (10-12 weeks), at 6 months, and at 12 month of follow-up from recruitment in order to capture acute and long-time impact of interventions. Hospitalizations and cardiovascular mortality will be a minimum of one year and a maximum of three year of follow-up in the cases recruited at the beginning of the study. The personnel assessing outcomes will be blinded to intervention assignment.

4.7. Analysis plan.

The analysis plan will include the following:

1. Baseline data analysis will be conducted in order to compare the distribution of sociodemographic and clinical characteristics of participants to test for homogeneity of groups with randomization.
2. Consider compliance with the rehabilitation study protocol at each center and in both arms,
3. Describe and compare participant adherence to the interventions (including voice calls),
4. Consider loss to follow-up to evaluate presence of bias, and eventually adjust in effectiveness analysis
5. An interim analysis for primary outcome in order to assess whether the trial is unlikely to show non-inferiority at the final analysis will be performed when 50% of the sample has been recruited and followed-up 1 year (46).
6. To test the primary hypothesis, recurrent cardiovascular events, differences in proportions will be estimated in absolute and relative ways as absolute risk difference (ARD) and relative risk (RR), respectively. Additionally, a survival analysis will be performed to estimate hazard ratio (HR).
    a. Both, intention to treat (ITT) and per protocol (PP) analysis will be considered since the ITT makes easier to established the non-inferiority and the PP is considered a more conservative approach. Non-inferiority will be considered established if both ITT and PP analyses support it (31).
    b. Analyses will be adjusted if unbalances in baseline characteristics are found
7. Depending on the results, subgroup analysis (e.g., sex) may be conducted to explain unexpected differences in outcomes
8. To test the secondary outcomes: For categorical outcomes both, ARD and RR will be estimated. For continuous outcomes, mean differences will be calculated.

4.8. Quality control

All processes involved in the study will be overseen. Specific actions are the following:

- Central management office: a central coordination center will be established, responsible for all general processes in the study such as securing and managing ethical and regulatory approvals, document and questionnaire preparation, field personnel training, measurement standardization, and data entry monitoring. The central coordinator will be responsible maintain regular communication with participating centers, solve problems and supervise the overall integrity of the study.
- Center coordinators: each center will have a local coordinator in charge of trial activities such as recruitment, random assignment, coordination of assessments, and sending data to the central office. The critical issues for which the coordinator is responsible include ensuring assignment concealment and blinding of outcome assessments.
- Monitoring: a professional with documented training in Good Clinical Practice (GCP) (47) will be engaged in order to monitor the conduct of the trial. The purpose will be to verify:
    - The protections of the rights and well-being of participants.
    - That the data obtained will be accurate, complete and checked against source documents.
    - That the study is consistent with the approved protocol, and is undertaken in accordance to the GCP guidelines and ethical requirements.

5.0 Ethical Issues

A number of treaties and declarations have been published, which outline the fundamental principles of ethical conduct in biomedical research. In the design of this proposal, the fulfillment of ethical principles of the Belmont Inform has been considered: the value of the research question, methodological rigor, investigators are scientifically-qualified, independent protocol evaluation, and plan that results will be published in a punctual and precise way. The specific bioethical values to be considered are:

- Respect for autonomy: all participants will decide freely on their enrolment after being given sufficient information and time to understand the information about the study; this will be demonstrated through signing an inform consent form.
- Beneficence: benefits and knowledge that will be gained has been considered, and is expected to be bigger than risks or discomforts. Additionally, considering the proven effectiveness of the standard exercise-based cardiac rehabilitation, the actual situation of exercise-based cardiac rehabilitation delivery in Chile, and the recent recommendations for an adapted model of cardiac rehabilitation for low-resources settings, the research design proposed is highly appropriate; indeed, the decision to use a non-inferiority active control study design instead of a superiority design is an ethical one. Specifically, this design is chosen when it would not be ethical to use a placebo, or a no-treatment control, because there is an effective treatment that provides an important benefit available to patients for the condition to be studied in the trial. In this manner, this non-inferiority trial represents a methodological contribution to reduce the exposure of patients only to usual care (48). More specifically, important aspects have been considered in this proposal (49):
    - Definition of non-Inferiority margin, based in the FDA recommendation.
    - Per protocol and intention to treat analysis.
    - Interim analysis.
    - Sample size adjustment.
- Not maleficence: in order to guarantee the participants protection, security and welfare, a research team and collaborators have been assembled to ensure the best quality trial from a clinical and methodological perspective.
- Justice: Participants' selection and recruitment will be performed in a non-discriminatory way, and based on eligibility criteria directly related to the study question. The randomization will guarantee that each participant will have the same probability of being assigned to one or the other group.

The study protocol and the Informed Consent form will be submitted to the corresponding Ethical Committees at the Sponsor Institution and each center where participants will be recruited. At the same time, the protocol will be registered in ClinicalTrials.gov.

6.0 Work Plan:

In the following diagram, a flowchart of the study with activities and the timeline expected is presented.

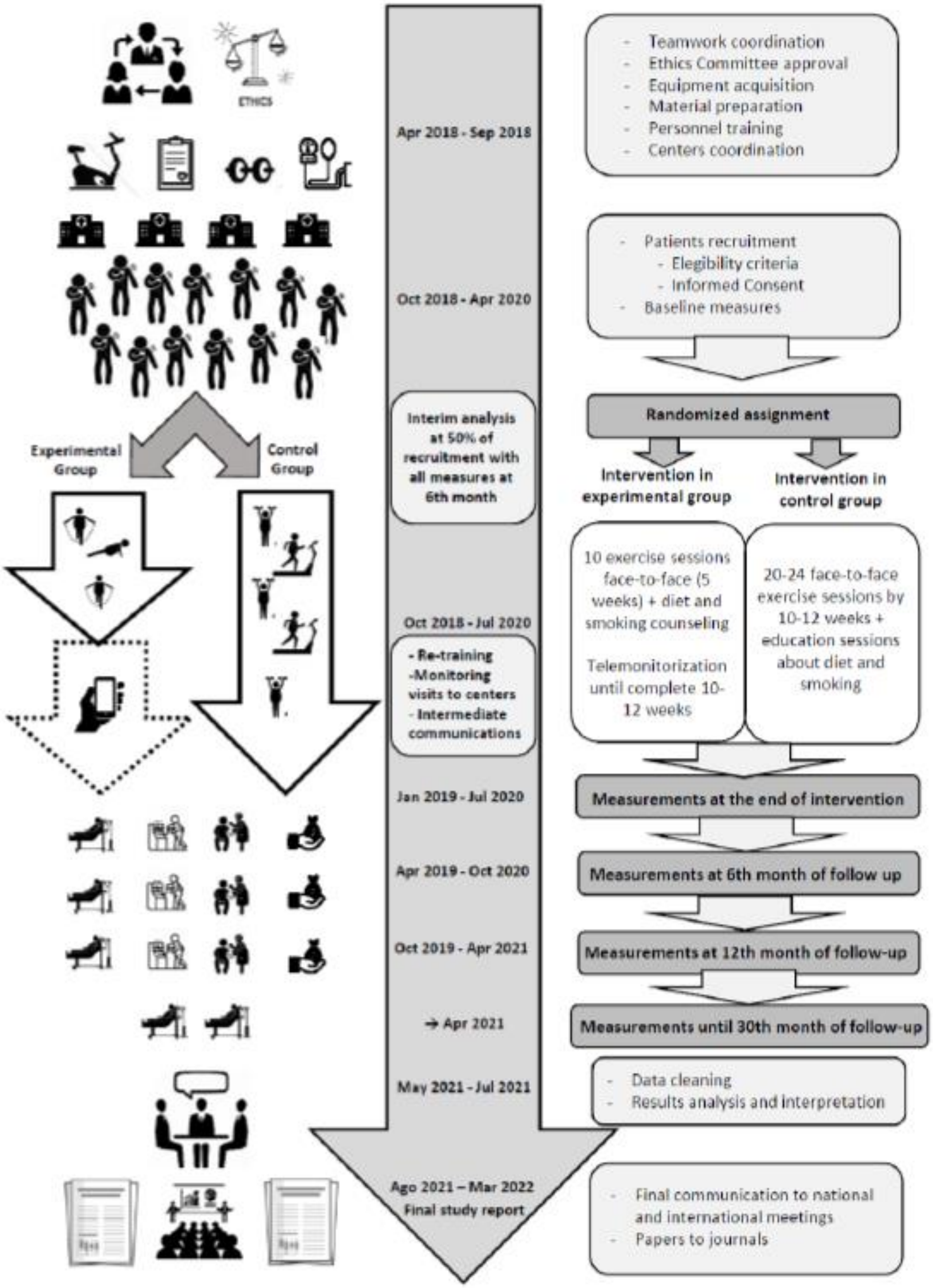

94

7.0 Work in progress:

7.1 Economical evaluation in Cardiac Rehabilitation: A cost-effectiveness study was performed (FONIS SA14ID0005) which evaluated three models of exercise-based cardiac rehabilitation through a Markov Model, concluding that cardiac rehabilitation is highly cost-effective for the public health system in Chile. The analysis of standard cardiac rehabilitation (as will be delivered in the control group in the current study) vs usual care showed an ICER of US$722.34, and when evaluated the Low-Resource Model of cardiac rehabilitation (service delivered as is recommended by ICCPR Consensus Statement, which is the intervention in the experimental group in this proposal) vs usual care the ICER was US$665.79. Is important to clarify that there was uncertainty in the estimation of this last model, arising from the fact that the effectiveness was not documented by clinical studies; this is an important justification for the present proposal.

7.2 Measurements standardization and training: Procedures to undertake the measurements included in this proposal have been locally standardized and validated. These procedures have now also been applied in other prospective studies (PURE and CESCAS). Investigators from this proposal have adapted, validated or/and developed the operating manuals for: event adjudication, physical measurements (blood pressure, weight, etc) (50,51), 6MWT (52), grip strength (35), IPAQ (38,53), FFQ (43,44), and EQ-5D-3L (FONIS SA14ID0005).

7.3 International Cooperation: Contact has been established with ICCPR, and the present chair who lead the development of the low-resource model for cardiac rehabilitation delivery, gave us details of the intervention that were inputs for the economic evaluation described above; she is available for cooperating in conducting this study.

95
96

8.0 Available Resources

8.1 For field work: We have a scientific team in epidemiologic and clinical studies, that at present is participating in the PURE cohort (3585 participants) and CESCAS cohort (1950 participants). There is also available trained professionals who will deliver the study interventions (physiotherapists), and others who will perform the outcomes measurements in the centers identified (i.e., in each center one coordinator who will be in charge of all processes involved has been identified). The centers have given an anticipate support for conducting the study (upon approval of all regulatory requirements), have capability to implement the interventions, and have cardiac rehabilitation demand that justifies the feasibility of recruitment of the required sample size for the study (around 500 patients per year, and more than 800 in the timeline estimated for recruitment).
The centers are: Complejo Hospitalario San José, Hospital San Juan de Dios, Hospital Regional de Antofagasta, Hospital Clínico de la Universidad de Chile, and Hospital Clínico San Borja Arriarán in Santiago, and Hospital Dr. Hernán Henríquez Aravena in Temuco.

8.2 Research team configuration: blend seniors with young researchers, and methodological and clinician expertise. We have experience in management of big scale studies, in statistical analyses, database construction, and data entry supervision.

97
98

BIBLIOGRAPHIC REFERENCES:

1. Murray CJL, Barber RM, Foreman KJ, Ozgoren AA, Abd-Allah F, Abera SF, et al. Global, regional, and national disability-adjusted life years (DALYs) for 306 diseases and injuries and healthy life expectancy (HALE) for 188 countries, 1990-2013: Quantifying the epidemiological transition. Lancet. 2015;386(10009):2145–91.
2. Lanas F, Serón P, Lanas A. Coronary heart disease and risk factors in Latin America. Vol. 8, Global Heart. 2013. p. 341–8.
3. Fernando L, Pamela S, Alejandra L. Cardiovascular disease in latin america: The growing epidemic. Prog Cardiovasc Dis. 2014;57(3):262–7.
4. Organización Panamericana de la Salud / Organización Mundial de la Salud. Salud en Sudamérica. Washington DC; 2012.
5. Bedregal P, Margozzi P, Gonzalez C et al. Informe Final Estudio de Carga de Enfermedad y Carga Atribuible. MINSAL.

99

6. Smith SC, Benjamin EJ, Bonow RO, Braun LT, Mark A, Franklin BA, et al. AHA / ACCF Guideline AHA / ACCF Secondary Prevention and Risk Reduction Therapy for Patients With Coronary and Other Atherosclerotic Vascular Disease : 2011 Update A Guideline From the American Heart Association and American College. 2011;
7. Steg PG, James SK, Atar D, Badano LP, Lundqvist CB, Borger MA, et al. ESC Guidelines for the management of acute myocardial infarction in patients presenting with ST-segment elevation. Eur Heart J. 2012;33(20):2569–619.
8. Teo K, Lear S, Islam S, Mony P, Dehghan M, Li W, et al. Prevalence of a Healthy Lifestyle Among Individuals With Cardiovascular Disease in High-, Middle- and Low-Income Countries. JAMA. 2013 Apr 17;309(15):1613.
9. Nazzal C, Lanas F, Bugueño C, Mercadal E, Garcés E, Garmendia ML, et al. Prevención secundaria post infarto agudo de miocardio en hospitales públicos: implementación y resultados de las garantías GES TT - Universal health coverage and accomplishment of secondary prevention goals among patients with acute myocardial infarction. Rev Med Chil. 2013;141(8):977–86.
10. O'Gara PT, Kushner FG, Ascheim DD, Casey DE, Chung MK, de Lemos JA, et al. 2013 ACCF/AHA Guideline for the Management of ST-Elevation Myocardial Infarction. Circulation. 2012;
11. Germany HG, Ireland IG, Verschuren WMM, Albus C, France PB, Denmark GB, et al. European Guidelines on cardiovascular disease prevention in clinical practice (version 2012) The Fifth Joint Task Force of the European Society of Cardiology. 2012;1635–701.
12. Thomas RJ, King M, Lui K, Oldridge N, Piña IL SJ. AACVPR/ACC/AHA 2007 performance measures on cardiac rehabilitation for referral to and delivery of cardiac rehabilitation/secondary prevention services. Circulation. 2007;116:1611–42.
13. Serón P, Lanas F, Ríos E, Bonfill X, Alonso-Coello P. Evaluation of the Quality of Clinical Guidelines for Cardiac Rehabilitation. J Cardiopulm Rehabil Prev. 2015;35(1):1–12.
14. Anderson L, Thompson DR, Oldridge N, Zwisler A-D, Rees K, Martin N, et al. Exercise-based cardiac rehabilitation for coronary heart disease. Taylor RS, editor. Cochrane Database Syst Rev. 2016 Jan 5;(1):CD001800.
15. Turk-Adawi K, Sarrafzadegan N, Grace SL. Global availability of cardiac rehabilitation. Nat Rev Cardiol. 2014 Jul 15;11(10):586–96.
16. Santibáñez C. Situación actual de la rehabilitación cardiaca en Chile. Rev médica .... 2012;561–8.
17. Cortes-Bergoderi M, Lopez-Jimenez F, Herdy AH, Zeballos C, Anchique C, Santibañez C, et al. Availability and characteristics of cardiovascular rehabilitation programs in South America. J Cardiopulm Rehabil Prev. 2013;33(1):33–41.
18. Wong WP, Feng J, Pwee KH, Lim J. A systematic review of economic evaluations of cardiac rehabilitation. BMC Health Serv Res. 2012;12(1):1.
19. Kühr EM, Ribeiro RA, Rohde LEP, Polanczyk CA. Cost-effectiveness of supervised exercise therapy in heart failure patients. Value Heal. 2011;14(5 SUPPL.):S100–7.
20. Rincón M, Rojas MX, Rodriguez Romero VA, Tamayo DC, Franco C, Castro H, et al. Economic Evaluation of Exercise-Based Cardiac Rehabilitation Programs for Chronic Heart Failure Patients in Colombia. J Cardiopulm Rehabil Prev. 2016;36(1):12–9.
21. Oldridge NB, Pakosh MT, Thomas RJ. Cardiac rehabilitation in low- and middle-income countries : a review on cost and cost-effectiveness. 2016;(July 2015):77–82.
22. Moghei M, Turk-Adawi K, Isaranuwatchai W, Sarrafzadegan N, Oh P, Chessex C, et al. Cardiac rehabilitation costs. 2017;
23. Grace SL, Turk-Adawi KI, Contractor A, Atrey A, Campbell NRC, Derman W, et al. Cardiac Rehabilitation Delivery Model for Low-Resource Settings: An International Council of Cardiovascular Prevention and Rehabilitation Consensus Statement. Prog Cardiovasc Dis. 2016;59:303–22.
24. Clark R a, Conway A, Poulsen V, Keech W, Tirimacco R, Tideman P. Alternative models of cardiac rehabilitation: a systematic review. Eur J Prev Cardiol. 2015;22(1).
25. Jelinek M, Vale MJ, Liew D, Grigg L, Dart A, Bm B, et al. The COACH Program Produces Sustained Improvements in Cardiovascular Risk Factors and Adherence to Recommended Medications—Two Years Follow-up. Hear Lung Circ. 2009;18:388–92.
26. Price KJ, Gordon BA, Bird SR, Benson AC. A review of guidelines for cardiac rehabilitation exercise programmes: Is there an international consensus? Eur J Prev Cardiol. 2016 Nov 27;23(16):1715–33.

27. Bjarnason-Wehrens B, McGee H, Zwisler A-D, Piepoli MF, Benzer W, Schmid J-P, et al. Cardiac rehabilitation in Europe: results from the European Cardiac Rehabilitation Inventory Survey. Eur J Cardiovasc Prev Rehabil. 2010 Aug;17(4):410–8.
28. Grace SL, Turk-adawi KI, Contractor A, Atrey A, Campbell N, Derman W, et al. Cardiac rehabilitation delivery model for low-resource settings. Heart. 2016;102:1449–55.
29. Kenneth F Schulz, Douglas G Altman, David Moher, for the CONSORT Group. CONSORT 2010 Statement: updated guidelines for reporting parallel group randomised trials. BMJ. 2010;340:698–702.
30. RIEBE D, FRANKLIN BA, THOMPSON PD, GARBER CE, WHITFIELD GP, MAGAL M, et al. Updating ACSM's Recommendations for Exercise Preparticipation Health Screening. Med Sci Sport Exerc. 2015 Nov;47(11):2473–9.
31. Walker E, Nowacki AS. Understanding Equivalence and Noninferiority Testing. J Gen Intern Med. 26(2):192–6.
32. Redfern J, Thiagalingam A, Jan S, Whittaker R, Hackett M, Mooney J, et al. Development of a set of mobile phone text messages designed for prevention of recurrent cardiovascular events. Eur J Prev Cardiol. 2014;21(4):492–9.
33. Pickering TG, Hall JE, Appel LJ, Falkner BE, Graves J, Hill MN, et al. Recommendations for Blood Pressure Measurement in Humans and Experimental Animals. Circulation. 2005;111(5).
34. Crapo RO, Casaburi R, Coates AL, Enright PL, MacIntyre NR, McKay RT, et al. ATS statement: Guidelines for the six-minute walk test. Am J Respir Crit Care Med. 2002;166(1):111–7.
35. Leong DP, Teo KK, Rangarajan S, Lopez-jaramillo P, Jr AA, Orlandini A, et al. Prognostic value of grip strength : fi ndings from the Prospective Urban Rural Epidemiology (PURE) study. Lancet. 2015;6736(14):1–8.
36. Roberts HC, Denison HJ, Martin HJ, Patel HP, Syddall H, Cooper C, et al. A review of the measurement of grip strength in clinical and epidemiological studies: Towards a standardised approach. Age Ageing. 2011;40(4):423–9.
37. CRAIG CL, MARSHALL AL, SJ??STR??M M, BAUMAN AE, BOOTH ML, AINSWORTH BE, et al. International Physical Activity Questionnaire: 12-Country Reliability and Validity. Med Sci Sport Exerc. 2003 Aug;35(8):1381–95.
38. Poggio R, Seron P, Calandrelli M, Ponzo J, Mores N, Matta MG, et al. Prevalence, Patterns, and Correlates of Physical Activity Among the Adult Population in Latin America. Glob Heart. 2016;11(1):81–8.
39. Serón P, Muñoz S, Lanas F. Nivel de actividad física medida a través del cuestionario internacional de actividad física en población chilena Levels of physical activity in an urban population from Temuco, Chile. artículo Investig rev Med chile. 2010;138:1232–9.
40. Martínez-González M a, Fernández-Jarne E, Serrano-Martínez M, Wright M, Gomez-Gracia E. Development of a short dietary intake questionnaire for the quantitative estimation of adherence to a cardioprotective Mediterranean diet. Eur J Clin Nutr. 2004;58(11):1550–2.
41. Schröder H, Fitó M, Estruch R, Martínez-González MA, Corella D, Salas-Salvadó J, et al. A short screener is valid for assessing Mediterranean diet adherence among older Spanish men and women. J Nutr. 2011;141(6):1140–5.
42. Facultad de medecina de la Universidad de Chile. Encuesta Nacional De Consumo Alimentario. 2010;21.
43. Elorriaga N, Irazola VE, Defagó MD, Britz M, Martínez-Oakley SP, Witriw AM, et al. Validation of a self-administered FFQ in adults in Argentina, Chile and Uruguay. Public Health Nutr. 2015 Jan 14;18(1):59–67.
44. Dehghan M, Martinez S, Zhang XH, Seron P, Lanas F, Islam S, et al. Relative validity of an FFQ to estimate daily food and nutrient intakes for Chilean adults. Public Health Nutr. 2013;16(10):1782–8.
45. Zarate V, Kind P, Valenzuela P, Vignau A, Olivares-Tirado P, Munoz A. Social valuation of EQ-5D health states: The chilean case. Value Heal. 2011;14(8):1135–41.
46. Bratton DJ, Williams HC, Kahan BC, Phillips PP, Nunn AJ. When inferiority meets non-inferiority : Implications for interim analyses. Clin TRIALS Clin Trials. 2012;9(9):605–9.

47. World Health Organization. HANDBOOK FOR GOOD CLINICAL RESEARCH PRACTICE (GCP). 2005.
48. Pinto VF. Non-inferiority clinical trials: concepts and issues. J Vasc Bras. 2010 Sep;9(3):145–51.
49. U.S. Department of Health and Human Services, Food and Drug Administration, Center for Drug Evaluation and Research (CDER), Center for Biologics Evaluation and Research (CBER). Non-Inferiority Clinical Trials to Establish Effectiveness. Guidance for Industry. 2016.
50. Rubinstein AL, Irazola VE, Calandrelli M, Elorriaga N, Gutierrez L, Lanas F, et al. Multiple cardiometabolic risk factors in the Southern Cone of Latin America: A population-based study in Argentina, Chile, and Uruguay. Int J Cardiol. 2015;183:82–8.
51. Teo K, Chow CK, Vaz M, Rangarajan S, Yusuf S. The Prospective Urban Rural Epidemiology (PURE) study: Examining the impact of societal influences on chronic noncommunicable diseases in low-, middle-, and high-income countries. Am Heart J. 2009;158(1):1–7.e1.
52. Seron P, Riedemann P, Munoz S, Doussoulin A, Villarroel P, Cea X, et al. Effect of inspiratory muscle training on muscle strength and quality of life in patients with chronic airflow limitation: a randomized controlled trial. Arch Bronconeumol. 2005;41(11):601–6.
53. Serón P, Muñoz S, Lanas F. Levels of physical activity in an urban population from Temuco, Chile. Rev Med Chil. 2010;138(10):1232–9.
