# Supplementary material for: Hybrid Cardiac Rehabilitation Program in a Low-Resource Setting: A Randomized Clinical Trial
Source: JAMA Netw Open. 2024 Jan 9;7(1):e2350301. doi: 10.1001/jamanetworkopen.2023.50301 (PMC10777264; doi:10.1001/jamanetworkopen.2023.50301)
Supplement: Supplement 2. — eMethods 1. Inclusion and Exclusion Criteria eTable 1. Study Centers, Location, Ethics Committee Approvals, and Amendments eMethods 2. Text Message and Phone Call Content in HYCARET eTable 2. Measurements for Each Outcome at Each Follow-Up Point According to the Assigned Group eTable 3. Between-Group Effects by Assessment Point in PP Analysis by the Level of Adherence (60%, 40%, 20%) to the Intervention (Binary Outcomes) eTable 4. Between-Group Effects by Assessment Point in PP Analysis by the Level of Adherence (60%, 40%, 20%) to the Intervention (Continuous Outcomes) eReference [file jamanetwopen-e2350301-s002.pdf]

Supplementary Online Content

Seron P, Oliveros MJ, Marzuca-Nassr GN, et al; HYCARET investigators. Hybrid cardiac rehabilitation program in a low-resource setting: a randomized clinical trial. *JAMA Netw Open*. 2024;7(1):e2350301.
doi:10.1001/jamanetworkopen.2023.50301

eMethods 1. Inclusion and Exclusion Criteria
eTable 1. Study Centers, Location, Ethics Committee Approvals, and Amendments
eMethods 2. Text Message and Phone Call Content in HYCARET
eTable 2. Measurements for Each Outcome at Each Follow-Up Point According to the Assigned Group
eTable 3. Between-Group Effects by Assessment Point in PP Analysis by the Level of Adherence (60%, 40%, 20%) to the Intervention (Binary Outcomes)
eTable 4. Between-Group Effects by Assessment Point in PP Analysis by the Level of Adherence (60%, 40%, 20%) to the Intervention (Continuous Outcomes)
eReference

This supplementary material has been provided by the authors to give readers additional information about their work.

© 2024 Seron P et al. *JAMA Network Open*.

eMethods 1. Inclusion and exclusion criteria

Participants were included if were ≥18 years old, with coronary artery disease, including acute coronary syndrome or stable coronary vessel disease diagnosed by angiography or a stress test. They had to have: (1) been treated medically or by thrombolysis, angioplasty, or revascularization surgery, (2) referred to CR between 2 weeks and 2 months from their event, diagnosis or procedure, (3) been able to attend the health center twice a week over three months, and (4) have access to a mobile phone.

The exclusion criteria were that the patient had a planned a cardiac procedure in the following 12 months, explicit contraindication to performing exercise based on American College of Sport Medicine guidelines[1], comorbidities that would interfere with the ability to engage in CR (such as dementia, blindness, deafness, serious mental illness, or frailty) or musculoskeletal condition that would preclude the patient from performing exercise.

Riebe D, Franklin BA, Thompson PD, et al. Updating ACSM's Recommendations for Exercise Preparticipation Health Screening. Med Sci Sports Exerc. 2015;47(11):2473-2479. doi:10.1249/MSS.0000000000000664

© 2024 Seron P et al. *JAMA Network Open*.

eTable 1. Study centers, location, ethics committee approvals, and amendments.

Center	Location	Ethical Approvals Protocol	Amendments approvals	
			Incorporation of specific quality of life questionnaire (HeartQoL) and return to work outcome.	Recruitment Detention, and sample size modification (93 participants per arm), and no conducting interim analysis.
Hospital Hernán Henríquez Aravena & Centro de Habilidades UFRO	Temuco. South of Chile	- Acta N°032_18 at SEC of *Universidad de La Frontera* (May 23, 2018)<br>- Acta N° 267 at SEC *Servicio de Salud Araucanía Sur* (November 07, 2018).	- Ord. N° 055_2019 of *Universidad de La Frontera* (December 09, 2019).<br>- *N° Folio* 244 at SEC *Servicio de Salud Araucanía Sur* (December 23, 2019)	- Ord N° 042_2021 of *Universidad de La Frontera* (July 01, 2021)<br>- *Oficio* N° 159 at SEC *Servicio de Salud Araucanía Sur* (June 17, 2021)
Hospital Clínico Universidad de Chile	Santiago. The metropolitan area of Chile	- Acta N°032_18 at SEC of *Universidad de La Frontera* (May 23, 2018)<br>- N° 47/2018 at SEC *Hospital Clínico de la Universidad de Chile (August 29, 2018)*	- Ord. N° 055_2019 of *Universidad de La Frontera* (December 09, 2019)<br>- Approval letter, October 23, 2019. SEC *Hospital Clínico de la Universidad de Chile*	- Ord N° 042_2021 of *Universidad de La Frontera* (July 01, 2021)<br>- Approval letter, May 12, 2021. SEC *Hospital Clínico de la Universidad de Chile*
Complejo Hospitalario San José	Santiago. The metropolitan area of Chile	- Acta N°032_18 at SEC of *Universidad de La Frontera* (May 23, 2018)<br>- N° 056/2018 at SEC *Servicio de Salud Metropolitano Norte* (August 29, 2018)	- Ord. N° 055_2019 of *Universidad de La Frontera* (December 09, 2019)	- Ord N° 042_2021 of *Universidad de La Frontera* (July 01, 2021)<br>- Approval letter, June 23, 2021. SEC *Servicio de Salud Metropolitano Norte*
Hospital San Borja Arriarán	Santiago. The metropolitan area of Chile	- Acta N°032_18 at SEC of *Universidad de La Frontera* (May 23, 2018)<br>- N° 92/6 at SEC *Servicio de Salud Metropolitano Central* (August 10, 2018)	- Ord. N° 055_2019 of *Universidad de La Frontera* (December 09, 2019)<br>- Certificate, Oct 22, 2019. SEC *Servicio de Salud Metropolitano Central*	- Ord N° 042_2021 of *Universidad de La Frontera* (July 01, 2021)<br>- Certificate, May 25, 2021. SEC *Servicio de Salud Metropolitano Central*
Hospital San Juan de Dios	Santiago. The metropolitan area of Chile	- Acta N°032_18 at SEC of *Universidad de La Frontera* (May 23, 2018)	- Ord. N° 055_2019 of *Universidad de La Frontera* (Deccember 09, 2019)	- Ord N° 042_2021 of *Universidad de La Frontera* (July 01, 2021)
Hospital Regional de Antofagasta	Antofagasta. North of Chile	- Acta N°032_18 at SEC of *Universidad de La Frontera* (May 23, 2018)	- Ord. N° 055_2019 of *Universidad de La Frontera* (December 09, 2019)	- Ord N° 042_2021 of *Universidad de La Frontera* (July 01, 2021)

SEC = Scientific Ethical Committee

© 2024 Seron P et al. *JAMA Network Open*.

eMethods 2. Text message and phone call content in HYCARET.

Telephone accompaniment was provided in the non-face-to-face (home-based) stage of the hybrid cardiac rehabilitation group. In this stage, the participant was encouraged through text messages (SMS or WhatsApp according to the participant's preference) and phone calls, to continue with their active and healthy lifestyle, in accordance with the counseling carried out in the face-to-face stage. This accompaniment was structured with three text messages per week for four weeks, and two text messages in weeks five and six. In addition, three telephone calls were made. The following table shows the distribution of messages and calls with their identifying number.

	DAY	MESSAGE	CALL
WEEK 1	M	11	1
	W	12	
	F	13	
WEEK 2	M	21	
	W	22	
	F	23	
WEEK 3	M	31	2
	W	32	
	F	33	
WEEK 4	M	41	
	W	42	
	F	43	
WEEK 5	T	54	3
	T		
WEEK 6	T	63	
	T	61	

Below are the messages used per week and a description of how to approach telephone calls in general and also how to respond to potential concerns:

© 2024 Seron P et al. *JAMA Network Open*.

TEXT MESSAGES

The text messages were organized into groups: milestones (MILS), physical activity actions (PA-A), physical activities benefits (PA-B), nutrition advice (NUTRI), and medication adherence (MED).

WEEK 1		
Depending on the time of day, start with a greeting.<br>Always include the [NICKNAME] in the message, either in the greeting or farewell.<br>End with a farewell.		
TYPE	ID	MESSAGE
MILS	11	[NICKNAME], you can continue to work step by step!!! Don't forget that physical activity is good for you! A minimum of 30 minutes a day of exercise will help you have more energy, recover and feel better!
NUTRI	12	[NICKNAME] remember that healthy eating provides you with all the nutrients your body needs. Eat plenty of raw or cooked vegetables, including them in salads, puddings, omelets, and soups (goal = 3 servings a day). In addition, if you eat whole foods, legumes and vegetables, you are providing fiber to your body, which will help improve your digestion and keep your cholesterol normal.
PA-A	13	[NICKNAME] spend less time sitting, and choose to walk. Don't forget that the goal is to get at least 30 minutes of walking and moderate-intensity activity in a day. It is possible to be more active at home!
CALL 1		INITIAL

WEEK 2		
Depending on the time of day, start with a greeting.<br>Always include the [NICKNAME] in the message, either in the greeting or farewell.<br>End with a farewell.		
TYPE	ID	MESSAGE
PA-B	21	[NICKNAME], if you do at least 150 minutes of moderate-intensity physical activity a week, in addition to improving your health, you will feel more active and happier! Don't forget to work towards this goal, use the Borg scale to help you dose your exercise.
NUTRI	22	Fish consumption is beneficial to keep your heart healthy because it has a high contribution of omega 3. Eat baked or grilled fish or canned fish (tuna or horse mackerel), in this case, do not forget to wash it before eating to eliminate excess sodium (salt) or prefer those reduced in sodium.
PA-A	23	[NICKNAME], Visit your friends and family, and choose to walk! Remember to do at least 30 minutes of walking and moderate activity during the day.

© 2024 Seron P et al. *JAMA Network Open*.

WEEK 3		
Depending on the time of day, start with a greeting.<br>Always include the [NICKNAME] in the message, either in the greeting or farewell.<br>End with a farewell.		
TYPE	ID	MESSAGE
MILS	31	[NICKNAME] we are starting the 3rd week. At home, on the go and in your free time, choose to WALK! Adding 10-minute periods will help you meet your goal of 150 minutes a week!
NUTRI	32	[NICKNAME], by preferring foods low in sodium or salt, you will be helping to keep your blood pressure normal. A good tip is to take the salt shaker off the table and use spices and condiments such as lemon, turmeric, ginger, vinegar, garlic, mint or oregano to prepare your meals, thus reducing salt intake.
PA-A	33	As a general rule, if you can walk normally at your usual pace, you can also climb flights of stairs at your usual pace. Remember to build at least 30 minutes of moderate-intensity activity into your day!
CALL 2		

WEEK 4		
Depending on the time of day, start with a greeting.<br>Always include the [NICKNAME] in the message, either in the greeting or farewell.<br>End with a farewell.		
TYPE	ID	MESSAGE
PA-B	41	Remember: people who engage in at least 150 minutes of moderate-intensity physical activity per week achieve better control of risk factors such as blood pressure, cholesterol and weight more easily.
NUTRI	42	Read and compare food labels and choose foods with fewer warning labels. If you avoid sugar, sugary drinks, juices and sweets, you will be able to maintain a healthy weight.
PA-A	43	[APODO], remember that your choices for spending more energy and being more active were: (consider the choice of physical activity modality made during the face-to-face phase counseling)!<br>Keep them over time.

© 2024 Seron P et al. *JAMA Network Open*.

WEEK 5		
Depending on the time of day, start with a greeting.<br>Always include the [NICKNAME] in the message, either in the greeting or farewell.<br>End with a farewell.		
TYPE	ID	MESSAGE
PA-B	54	Don't forget that physical activity is good for you! A minimum of 30 minutes a day of exercise will help you improve your physical capacity, which means maintaining or improving your functionality!
MED		Taking your medications as directed by your doctor helps you control your cardiovascular risk factors. This, along with being physically active, eating healthy and not smoking will decrease your chance of suffering a future cardiac event.
CALL 3		

WEEK 6		
Depending on the time of day, start with a greeting.<br>Always include the [NICKNAME] in the message, either in the greeting or farewell.<br>End with a farewell.		
TYPE	ID	MESSAGE
AF-A	63	Remember that the goal is 150 minutes of physical activity per week, if you need to take public transportation, try to get off a little earlier and take advantage of walking briskly! Remember that you can put together periods of 10 minutes and achieve it!
MILS	61	"[[NICKNAME], you have reached the end of your Cardiac Rehabilitation program. Over the past few months you have been incorporating a healthier lifestyle: you are being more physically active, eating better and taking your medications. The time has come to demonstrate that you have made an independent and permanent commitment to your cardiovascular health. We will be contacting you for your follow-up evaluations.<br>Best regards, HYCARET team."

Finally, don't forget:

- Depending on the time of day start with a greeting.
- Always include the [NICKNAME] in the message, either in the greeting or farewell.
- End with a farewell.
- If the participant replies to you, send a reply of reception back, ok, understood, emoticon.

If the participant begins to ask more in-depth clinical questions respond with the following sample message, depending on the week of follow-up.

© 2024 Seron P et al. *JAMA Network Open*.

	Type of inquiry	Consideration	Programmed response
Week 1-3-5	Alarm Sign	If you have not yet made the phone call for this week, depending on your availability, suggest that the participant call you during the day.	[NICKNAME] according to what you tell me, I think it is important that you can visit your doctor or an Emergency Department.<br>If you would like, I could call you at about XX:XX hrs.
		If you have already made the call for the week, do not make it again.	[NICKNAME] Based on what you tell me, I think it is important for you to be able to visit your doctor or an emergency room.
	General inquiries	If you have not yet made the phone call for this week, depending on your availability, propose to the participant to call you during the day or the next day.	[NICKNAME] to better answer your questions, do you think I can call you (e.g. tomorrow at XX:XX hrs.?)
Week 2-4-6	Alarm Sign		[NICKNAME] Based on what you tell me, I think it is important for you to be able to visit your doctor or an emergency room.
	General inquiries		[NICKNAME] write down your questions, because next week I will contact you and we can talk about them.

PHONE CALLS

1. CALLS	Call by your [NICKNAME] and confirm full name
2. PRESENTATION	My name is ________________, I am calling you to continue your accompaniment as part of your Cardiac Rehabilitation program because when you were discharged from HHHA you were invited to participate in a study that seeks to evaluate the effectiveness of text messages and phone calls in meeting the worldwide recommendation for physical activity.
3. CHECK RECEIPT OF MESSAGES	First, I would like to corroborate that you are receiving the text messages (3 or 1 a week as appropriate) that we have been sending to your CELL PHONE or WHATSAPP (as appropriate).<br>* if yes continue to step 4.<br>** If no:<br>- Corroborate the type of message (text or WhatsApp) you have been sending, with the option expressed by the participant.

© 2024 Seron P et al. *JAMA Network Open*.

	- Corroborate that the number that should receive the message is the number you are calling.<br>- Send a test message and check that it arrives.<br>- If you identify that the participant does not know or understand how to open the messages, suggest that they ask someone they trust for help, and call back within 24 hours and recheck this step.<br>– If the problem is still not solved, suggest that the participant try to ask for assistance during the week, tell him/her that you will continue to send the messages, and continue with step 4.
4. REMEMBER THE RECOMMENDATION	The World Health Organization (WHO) recommendation for adults 18 years of age or older is: to dedicate 150 minutes per week to moderate aerobic physical activity. The activity should be practiced in sessions of at least 10 minutes.<br>Remember that you can do 3 walks of 10 minutes a day<br>2 of 15 minutes or one of 30 minutes.<br>In terms of nutrition, remember the importance of a balanced and cardioprotective diet, eating vegetables, fruit, fish, white meat, vegetable oils, pulses and foods rich in fibre. Depending on the week, we should reinforce the importance of low salt intake, the food labelling system and how to limit sugary foods.<br>Listen to the participant.
5. POSITIVELY MOTIVATE	Ask the participant about what he/she has done to fulfill the recommendation and listen to him/her.<br>* If he/she complies with the recommendation:<br>- Congratulate him/her.<br>- He/she needs to continue to do so and choose to walk in his/her free time, for transportation and at home.<br>- Remember that being physically active and follow a healthy diet will allow you to recover better, helps in the control of cardiovascular risk factors such as overweight, cholesterol, blood pressure and glycemia. In addition, your physical capacity will improve, that is, you will be less tired, your bones and muscles will be healthier and you will be able to perform your daily activities in a better way.<br>* If there are no problems in complying with the recommendation, listen to the participant, and guide solutions.
6. ANSWER QUESTION<br>7. GUIDE SOLUTIONS	Do you have any questions
8. FAREWELL	Thank you for your time, we will keep in touch.

© 2024 Seron P et al. *JAMA Network Open*.

MENSAJES DE TEXTO Y LLAMADAS TELEFÓNICAS EN HYCARET

LINIAMIENTOS GENERALES

El acompañamiento telefónico se realizó en la etapa no presencial (basada en el hogar) del grupo con rehabilitación cardiaca híbrida. En esta, el participante fue incentivado a través de mensajes de texto (SMS o WhatsApp según preferencia del participante) y llamadas telefónicas, para continuar con su estilo de vida activo y saludable, en concordancia con la consejería realizada en la etapa presencial. Este acompañamiento se estructuró con tres mensajes de texto semanales durante cuatro semanas, y dos mensajes de texto en las semanas cinco y seis. Adicionalmente se realizaron tres llamadas telefónicas. La siguiente tabla muestra la distribución de mensajes y llamadas con su número identificador.

SEMANA	DÍA	MENSAJE	LLAMADA
SEM 1	L	11	1
	M	12	
	V	13	
SEM 2	L	21	
	M	22	
	V	23	
SEM 3	L	31	2
	M	32	
	V	33	
SEM 4	L	41	
	M	42	
	V	43	
SEM 5	M	54	3
	J		
SEM 6	M	63	
	J	61	

A continuación, se muestran los mensajes utilizados por semana y se describe el abordaje de las llamadas telefónicas de manera general y también cómo responder ante potenciales dudas:

© 2024 Seron P et al. *JAMA Network Open*.

MENSAJES DE TEXTO

Los mensajes de texto se organizaron en grupos: hitos (HITOS), acciones de actividad física (AF-A), beneficio de la actividad física (AF-B), consejos de nutrición (NUTRI) y adherencia a la medicación (MED).

SEMANA 1		
Según el momento del día comenzar con un saludo.<br>Siempre incluir el [APODO] en el mensaje, ya sea en el saludo o despedida<br>Finalizar con una despedida.		
TIPO	ID	MENSAJE
HITO-B	11	[APODO], usted puede continuar trabajando paso a paso!! No olvide que la actividad física es buena para usted! Un mínimo de 30 minutos diarios de ejercicio le ayudarán a tener más energía, recuperarse y sentirse mejor!
NUTRI	12	[APODO] recuerde que una alimentación saludable le entrega todos los nutrientes que su cuerpo necesita. Consuma verduras crudas o cocidas en abundante cantidad, inclúyalas en ensaladas, budines, tortillas y sopas (meta= 3 porciones al día). Además, si usted consume alimentos integrales, legumbres y verduras, está aportando fibra a su cuerpo, que le ayudarán a mejorar su digestión y a mantener su colesterol normal.
AF-A	13	[APODO], dedique menos tiempo a estar sentado/a, y decida caminar. No olvide que la meta es realizar por lo menos 30 minutos de caminata y actividades de moderada intensidad en el día. Es posible ser más activo en casa!
LLAMADA 1		INICIAL

SEMANA 2		
Según el momento del día comenzar con un saludo.<br>Siempre incluir el [APODO] en el mensaje, ya sea en el saludo o despedida<br>Finalizar con una despedida.		
TIPO	ID	MENSAJE
AF-B	21	[APODO], si usted realiza por lo menos 150 minutos de actividad física de intensidad moderada a la semana, además de mejorar su salud, se sentirá más activo y feliz! No olvide trabajar por esta meta, use la escala de Borg que le ayudará a dosificar su ejercicio.
NUTRI	22	El consumo de pescado es beneficioso para mantener su corazón sano porque tiene un alto aporte de omega 3. Coma pescado al horno, a la plancha o también consúmalo enlatado (atún o jurel), en este caso no olvide lavarlo antes de consumir para eliminar el exceso de sodio (sal) o preferir los reducidos en sodio.
AF-A	23	[APODO], Visite a sus amigos y familiares, y escoja caminar! Recuerde realizar por lo menos 30 minutos de caminata y actividades moderadas en el día.

© 2024 Seron P et al. *JAMA Network Open*.

SEMANA 3		
Según el momento del día comenzar con un saludo.<br>Siempre incluir el [APODO] en el mensaje, ya sea en el saludo o despedida<br>Finalizar con una despedida.		
TIPO	ID	MENSAJE
HITO-B	31	[APODO], vamos comenzando la 3° semana. En su casa, para movilizarse y en su tiempo libre, escoja CAMINAR! Sumar periodos de 10 minutos, le ayudarán a cumplir la meta de 150 minutos a la semana!
NUTRI	32	[APODO], al preferir alimentos bajos en sodio o sal, estará ayudando a mantener su presión arterial normal, un buen consejo es sacar el salero de la mesa y usar especias y condimentos como limón, cúrcuma, jengibre, vinagre, ajo, menta u orégano para preparar sus comidas, así disminuirá el aporte de sal.
AF-A	33	Como regla general, si usted puede caminar normalmente a su ritmo habitual, también puede subir tramos de escaleras a su ritmo habitual. Recuerda acumular por lo menos 30 minutos de actividades de moderada intensidad en el día!
SEGUNDO LLAMADO		

SEMANA 4		
Según el momento del día comenzar con un saludo.<br>Siempre incluir el [APODO] en el mensaje, ya sea en el saludo o despedida<br>Finalizar con una despedida.		
TIPO	ID	MENSAJE
AF-B	41	Recuerda: las personas que realizan por lo menos 150 minutos de actividad física de intensidad moderada a la semana, logran un mejor control de los factores de riesgo como presión arterial, colesterol y peso con mayor facilidad.
NUTRI	42	Lea y compare las etiquetas de los alimentos y prefiera los que tengan menos sellos de advertencia. Si usted evita el azúcar, bebidas, jugos azucarados y dulces, podrá mantener un peso saludable.
AF-A	43	[APODO], recuerde que sus opciones elegidas para gastar más energía y ser más activo fueron: [*incluir las que haya elegido en la consejería*]! Manténgalas en el tiempo.

© 2024 Seron P et al. *JAMA Network Open*.

SEMANA 5		
Según el momento del día comenzar con un saludo.<br>Siempre incluir el [APODO] en el mensaje, ya sea en el saludo o despedida<br>Finalizar con una despedida.		
TIPO	ID	MENSAJE
AF-B	54	No olvide que la actividad física es buena para usted! Un mínimo de 30 minutos diarios de ejercicio le ayudarán a mejorar su capacidad física, lo que significa mantener o mejorar su funcionalidad!
MED		Tomar sus medicamentos según la indicación de su médico le ayuda a controlar sus factores de riesgo cardiovascular. Esto, junto con ser físicamente activo, alimentarse saludablemente y no fumar disminuirán su posibilidad de que sufra un futuro evento cardiaco.
TERCER LLAMADO		

SEMANA 6		
Según el momento del día comenzar con un saludo.<br>Siempre incluir el [APODO] en el mensaje, ya sea en el saludo o despedida<br>Finalizar con una despedida.		
TIPO	ID	MENSAJE
AF-A	63	Recuerde que la meta son 150 minutos de actividad física a la semana, si necesita transportarse en locomoción colectiva, intente bajarse un poco antes y aproveche de caminar a paso rápido! Recuerde que puede juntar periodos de 10 minutos y lograrlo!
HITO-B	61	"[APODO], ha llegado al final de su programa de Rehabilitación Cardíaca. En estos últimos meses usted ha ido incorporando un estilo de vida más saludable: está siendo más activo físicamente, se está alimentando mejor y está tomando sus medicamentos. Ha llegado el momento de demostrar que se ha comprometido con su salud cardiovascular de manera independiente y permanente.<br>Lo estaremos contactando para sus evaluaciones de seguimiento.<br>Un abrazo, equipo HYCARET."

NO OLVIDAR:

- Según el momento del día comenzar con un saludando.
- Siempre incluir el [APODO] en el mensaje, ya sea en el saludo o despedida
- Finalizar con una despedida.
- Si el participante le responde, envíe una respuesta de recepción de vuelta, ok, entendido, emoticón, etc.
- Si el participante comienza a realizar preguntas clínicas de mayor profundidad responder con el siguiente mensaje tipo, según la semana de seguimiento.

© 2024 Seron P et al. *JAMA Network Open*.

	Tipo de consulta	Consideración	Respuesta programada
Semana 1-3-5	Signo de Alarma	Si usted aún no ha realizado el llamado telefónico que corresponde a esta semana, según su disponibilidad propóngale al participante llamarlo durante el día.	[APODO] según lo que me cuenta, creo que es importante que pueda visitar a su médico o un servicio de Urgencia.<br>Si le parece podría llamarlo como a las XX:XX hrs.
		Si usted ya realizó la llamada correspondiente a la semana, no vuelva a realizarla.	[APODO] según lo que me cuenta, creo que es importante que pueda visitar a su médico o un servicio de Urgencia.
	Consultas generales	Si usted aún no ha realizado el llamado telefónico que corresponde a esta semana, según su disponibilidad propóngale al participante llamarlo durante el día o día siguiente.	[APODO] para responder de mejor manera a sus dudas, le parece que pueda llamarlo (Ej: mañana a las XX:XX hrs.?)
Semana 2-4-6	Signo de Alarma		[APODO] según lo que me cuenta, creo que es importante que pueda visitar a su médico o un servicio de Urgencia.
	Consultas generales		[APODO] anote sus dudas, porque la próxima semana me contactaré con usted y podremos conversar respecto de ellas.

SIGNOS DE ALARMA

- Dolor anginoso (real)
- Pérdida del conocimiento.
- Síncope
- Dificultad aguda respiratoria.

© 2024 Seron P et al. *JAMA Network Open*.

LLAMADAS TELEFONICAS

1. SALUDO	Llamar por su [APODO] y confirmar nombre completo
2. PRESENTACIÓN	Mi nombre es ________________, lo estoy llamando para continuar con su acompañamiento como parte de su programa de Rehabilitación Cardiaca, porque a Ud cuando fue dado de alta del HHHA se le invitó a participar en un estudio que busca evaluar la efectividad de los mensajes de texto y llamadas telefónicas en el cumplimiento de la recomendación mundial de actividad física
3. CORROBORAR RECEPCIÓN DE MENSAJES	Primero me gustaría corroborar que usted está recibiendo los mensajes de texto (3 o 1 a la semana según corresponda) que hemos estado enviando a su CELULAR o WHATSAPP (según corresponda)<br>* si dice que sí continúe con el paso 4.<br>** si dice que no:<br>– Corrobore el tipo de mensaje (texto o whatsapp) que ha estado enviando, con la opción que expresa el participante.<br>– Corrobore que el número que debe recibir el mensaje, es el número al que está llamando.<br>– Envíe un mensaje de prueba y corroboré que llega.<br>– Si identifica que el participante no sabe o no entiende como abrir los mensajes, sugiera que pida ayuda a alguien de confianza, y vuelva llamar dentro de las próximas 24 horas y vuelva a corroborar este paso.<br>– Si aun así el problema no se soluciona, sugiera al participante que intente pedir asistencia durante la semana, coméntele que le seguirán enviando los mensajes y continúe con el paso 4.
4. RECORDAR LA RECOMENDACIÓN	La recomendación de la Organización Mundial de la Salud (OMS), para adultos de 18 años o más es: dedicar 150 minutos semanales a realizar actividades físicas moderadas aeróbicas. La actividad debe practicarse en sesiones de 10 minutos, como mínimo.<br>Recordar que puede realizar 3 caminatas de 10 minutos al día<br>2 de 15 min o una de 30 minutos.<br>En relación a lo nutricional recordar la importancia de tener una dieta equilibrada y cardioprotectora, consumiendo verduras, frutas, pescado, carnes blancas, aceites vegetales, legumbres y alimentos ricos en fibra. Dependiendo de la semana ir reforzando sobre el consumo de sal bajo, el sistema de etiquetado de los alimentos y cómo, limitar los alimentos azucarados<br>Escuchar al participante.
5. MOTIVAR POSITIVAMENTE	Preguntar al participante sobre qué ha hecho para cumplir la recomendación y escucharlo.

© 2024 Seron P et al. *JAMA Network Open*.

	* Si cumple la recomendación:<br>– Felicítelo<br>– Es necesario que continúe haciéndolo y escoja caminar en su tiempo libre, para transportarse y en su casa.<br>– Recuerde que ser activo físicamente y llevar una dieta saludable le permitirá recuperarse mejor, ayuda en el control de los factores de riesgo cardiovascular como sobrepeso, colesterol, presión arterial y glicemia. Además su capacidad física mejorará, es decir se cansará menos, sus huesos y músculos estarán más saludables por lo que podrá realizar sus actividades diarias de mejor manera.<br>* Si no c inconvenientes para cumplir la recomendación, escuche al participante, y oriente soluciones.
6. CONTESTAR DUDA<br>7. ORIENTAR SOLUCIONES	Tiene alguna duda
8. Despedida	Gracias por su tiempo, seguiremos en contacto.

© 2024 Seron P et al. *JAMA Network Open*.

eTable 2. Measurements for each outcome at each follow-up point according to the assigned group.

	END OF INTERVENTION		6th MONTHS		12th MONTHS	
	HYBRID CR	STANDARD CR	HYBRID CR	STANDARD CR	HYBRID CR	STANDARD CR
**PRIMARY OUTCOMES**	**n=93**	**n=98**	**n=74**	**n=65**	**n=80**	**n=77**
Recurrent cardiovascular event						
Fatal cardiovascular events (%)	1 (1.07%)	1 (1.02%)	0	0	1 (1.25%)	0
Non-fatal Cardiovascular events (%)	1 (1.07%)	2 (2.04%)	2 (2.70%)	3 (4.61%)	2 (2.5x%)	4 (5.19%)
Hospitalization (%)	0	1 (1.02%)	1 (1.35%)	2 (3.08%)	1 (1.25%)	3 (3.89%)
Cardiovascular Events Composite (%)	1 (1.07%)	2 (2.04%)	2 (2.70%)	3 (4.61%)	2 (2.5x%)	4 (5.19%)
**SECONDARY OUTCOMES (QUESTIONNAIRES)**	**n=66**	**n=63**	**n=63**	**n=55**	**n=80**	**n=78**
Health-related quality of life						
HeartQoL-global*, mean, SD	2.61 ± 0.06	2.56 ± 0.06	2.53 ± 0.06	2.58 ± 0.05	2.58 ± 0.06	2.54 ± 0.06
HeartQoL-physical function, mean, SD	2.78 ± 0.05	2.70 ± 0.06	2.6 ± 0.04	2.66 ± 0.05	2.75 ± 0.06	2.63 ± 0.07
HeartQoL-emotional function, mean, SD	2.53 ± 0.05	2.48 ± 0.07	2.45 ± 0.06	2.54 ± 0.05	2.49 ± 0.07	2.49 ± 0.07
EQ-5D, VASS**, mean, SD	77.65 ± 17.89	75.43 ± 15.69	77.05 ± 2.61	76.6 ± 2.58	73.92 ± 2.14	73.85 ± 1.94
Adherence to lifestyle recommendations						
PA recommendations (%)	62 (93.94%)	55 (87.3%)	74 (92.50%)	65 (81.25%)	58 (93.55%)	50 (89.29%)
Diet recommendations (%)	56 (92.42%)	55 (88.71%)	58 (95.24%)	47 (87.04%)	72 (90.00%)	68 (88.31%)
Return-to-work, days, mean, SD***	82.17 ± 101.69	76.92 ± 84.77	-	-	-	-
**SECONDARY OUTCOMES (PHYSICAL MEASUREMENTS)**	**n=50**	**n=48**	**n=25**	**n=21**	**n=11**	**n=12**
Functional capacity in 6MWT, mts, mean,SD	555.26 ± 131.48	529.13 ± 122.9	554.48 ± 79.78	513.90 ± 102.57	510±0.00	495.00 ± 63.63
Muscle Strength, Kg, mean, SD	16.30 ± 4.78	17.31 ± 4.44	17.488 ± 5.47	16.81 ± 5.45	13±0.00	19.50 ± 2.12

CR: Cardiac Rehabilitation; SD=Standard Deviation; HeartQoL=Heart quality of life measure; EQ-5D=EuroQol 5 dimensions; VAS=Visual Analogue Scale; PA=Physical Activity; 6MWT=6 Minute Walk Test; mts=meters; Kg=Kilograms.
** Scale from 0 to 3, where 0 very much affected, 1 quite affected, 2 slightly affected, 3 not at all affected; ** Vertical visual scale ranged from 0 to 100, where 0 indicates the worst state of health imagined by the participant and 100 the best state of health; *** Was considered at the end of the follow-up.*
-not assessed at this time point.

© 2024 Seron P et al. *JAMA Network Open*.

eTable 3. Between-group effects by assessment point in PP analysis by the level of adherence (60%, 40%, 20%) to the intervention (binary outcomes)

	END OF INTERVENTION		6 MONTHS		12 MONTHS	
	**ARD (CI 95%)**	**RR (CI 95%)**	**ARD (CI 95%)**	**RR (CI 95%)**	**ARD (CI 95%)**	**RR (CI 95%)**
**PP ANALYSIS. AT LEAST 60% ADHERENCE TO SUPERVISED SESSIONS (Hybrid CR arms, n=76 / Standard CR arm, n=51).**						
**Recurrent cardiovascular Events**	NAT	NAT	NAT	NAT	0.01 (-0.06 – 0.09)	1.34 (0.25 – 7.06)
**Adherence to physical activity recommendations**	0.04 (-0.06-0.14)	1.04 (0.93-1.2)	0.07 (-0.04-0.19)	1.08 (0.95-1.24)	0.04 (-0.09-0.16)	1.04 (0.90- 0.20)
**Adherence to diet recommendations**	0.03 (-0.15-0.09)	0.75 (0.23-2.42)	0.06 (-0.18-0.06)	0.49 (0.12-2.08)	0.05 (-0.16-0.05)	0.52 (0.15-1.84)
**PP ANALYSIS. AT LEAST 40% ADHERENCE TO SUPERVISED SESSIONS (Hybrid CR arms, n=79 / Standard CR arm, n=69).**						
**Recurrent cardiovascular Events**	NAT	NAT	NAT	NAT	0.01 (-0.07 – 0.08)	1.09 (0.31 – 0.30)
**Adherence to physical activity recommendations**	0.08 (-0.03-0.18)	1.09 (0.97-1.22)	0.09 (-0.02-0.20)	1.11 (0.98- 1.26)	0.03 (-0.08-0.15)	1.04 (0.91-1.18)
**Adherence to diet recommendations**	0.04 (-0.16-0.07)	0.65 (0.22-1.94)	0.08 (-0.19-0.04)	0.41 (0.11-1.54)	0.05 (-0.14-0.05)	0.54 (0.16- 1.84)
**PP ANALYSIS. AT LEAST 20% ADHERENCE TO SUPERVISED SESSIONS (Hybrid CR arms, n=86 / Standard CR arm, n=81).**						
**Recurrent cardiovascular Events**	NAT	NAT	NAT	NAT	0.00 (-0.07 – 0.07)	0.91 (0.28 – 3.13)
**Adherence to physical activity recommendations**	0.09 (-0.01-0.19)	1.11 (0.98-1.24)	0.14 (0.03- 0.25)	1.17 (1.03-0.34)	0.05 (-0.06-0.16)	1.06 (0.94-0.16)
**Adherence to diet recommendations**	0.04 (-0.15-0.06)	0.65 (0.22-1.93)	0.09 (-0.20-0.02)	0.35 (0.09-1.29)	0.02 (-0.12-0.09)	0.86 (0.34-2.16)

ITT, intention to treat; PP, per protocol; ARD, absolute risk difference; RR, relative risk; CI, confidence interval; CR, cardiac rehabilitation; NAT= not assessed at this time point

© 2024 Seron P et al. *JAMA Network Open*.

eTable 4. Between-group effects by assessment point in PP analysis by the level of adherence (60%, 40%. 20%) to the intervention (continuous outcomes)

	END OF INTERVENTION	6 MONTHS	12 MONTHS
	**Mean Difference (CI 95%)**	**Mean Difference (CI 95%)**	**Mean Difference (CI 95%)**
**PP ANALYSIS. AT LEAST 60% ADHERENCE TO SUPERVISED SESSIONS (Hybrid CR arms, n=76 / Standard CR arm, n=51).**			
**HeartQoL, global**	-0.01 (-0.21 - 0.20)	0.10 (-0.061 - 0.26)	-0.002 (-0.18 - 0.18)
**HeartQoL, physical function**	-0.40 (-0.23 - 0.15)	0.01 (-0.17 - 0.19)	-0.10 (-0.29 - 0.09)
**HeartQoL, emotional function**	0.01 (-0.22 - 0.24)	0.14 (-0.03 - 0.32)	0.05 (-0.15 - 0.26)
**EQ-5D, VAS**	ISS	-0.46 (-2.21 – 1.99)	-1.34 (-2.43 – 0.32)
**Exercise capacity, mts**	-16.13 (-71.72 - 39.46)	ISS	ISS
**Muscle strength, kg**	ISS	ISS	ISS
**Return-to-work**	NAT	NAT	NAT
**PP ANALYSIS. AT LEAST 40% ADHERENCE TO SUPERVISED SESSIONS (Hybrid CR arms, n=79 / Standard CR arm, n=69).**			
**HeartQoL, global**	-0.09 (-0.28 - 0.10)	0.08 (-0.07 - 0.23)	-0.70 (-0.25 - 0.11)
**HeartQoL, physical function**	-0.09 (-0.27 - 0.08)	0.02 (-0.14 - 0.18)	-0.17 (-0.35 - 0.01)
**HeartQoL, emotional function**	-0.09 (-0.31 - 0.13)	0.11 (-0.056 - 0.28)	-0.01 (-0.21 - 0.18)
**EQ-5D, VAS**	-2.01 (-8.21 - 4.20)	-1.54 (-2.79 – 1.11)	-0.13 (-1.65 – 1.52)
**Exercise capacity, mts**	-26.12 (-78.59 - 26.34)	ISS	ISS
**Muscle strength, kg**	1.02 (-0.91 - 2.95)	ISS	ISS
**Return-to-work**	NAT	NAT	NAT

© 2024 Seron P et al. *JAMA Network Open*.

	END OF INTERVENTION	6 MONTHS	12 MONTHS
	**Mean Difference (CI 95%)**	**Mean Difference (CI 95%)**	**Mean Difference (CI 95%)**
**PP ANALYSIS. AT LEAST 20% ADHERENCE TO SUPERVISED SESSIONS (Hybrid CR arms, n=86 / Standard CR arm, n=81).**			
**HeartQoL, global**	(-) 0.09 (-0.27 - 0.08)	0.003 (-0.15 - 0.15)	(-) 0.08 (-0.25 - 0.09)
**HeartQoL, physical function**	(-) 0.11 (-0.27 - 0.05)	(-) 0.06 (-0.23 - 0.10)	(-) 0.15 (-0.33 - 0.02)
**HeartQoL, emotional function**	(-) 0.08 (-0.29 - 0.12)	0.04 (-0.12 - 0.20)	(-) 0.04 (-0.23 - 0.15)
**EQ-5D, VAS**	-2.39 (-8.51 - 3.72)	-1.15 (-2.32 – 1.01)	-1.35 (-2.56 – 0.97)
**Exercise capacity, mts**	-26.12 (-78.59 - 26.34)	ISS	ISS
**Muscle strength, kg**	1.02 (-0.91 - 2.95)	ISS	ISS
**Return-to-work**	NAT	NAT	NAT

PP, per protocol; Qol, quality of life; EQ-5D=EuroQol 5 dimensions; VAS=Visual Analogue Scale; CI, confidence interval; mts=meters; Kg=Kilograms; ISS=insufficient sample size; NAT= not assessed at this time point

© 2024 Seron P et al. *JAMA Network Open*.

eReference

1. Riebe D, Franklin BA, Thompson PD, et al. Updating ACSM's Recommendations for Exercise Preparticipation Health Screening. *Med Sci Sports Exerc* 2015;47(11):2473-2479. doi:10.1249/MSS.0000000000000664

© 2024 Seron P et al. *JAMA Network Open*.
