# Supplementary material for: Hybrid Cardiac Rehabilitation Program in a Low-Resource Setting: A Randomized Clinical Trial
Source: JAMA Netw Open. 2024 Jan 9;7(1):e2350301. doi: 10.1001/jamanetworkopen.2023.50301 (PMC10777264; doi:10.1001/jamanetworkopen.2023.50301)
Supplement: Supplement 3. — HYCARET Investigators [file jamanetwopen-e2350301-s003.pdf]

Supplemental Online Content: Nonauthor Collaborators
*First name, last name, and suffix (if applicable) are required and will appear in PubMed.

*Group Name(s): HYCARET Investigators							
***First Name and Middle Initial(s)**	***Last Name**	***Suffix (eg, Jr, III)**	Academic Degrees	Institution	Location (city, state/province, country)	Role or Contribution, eg, chair, principal investigator	Group (if more than 1 Group listed in the byline) and/or Subgroup (eg, Steering Committee)
Daniela	Gomez		PhD	Universidad de La Frontera	Temuco, Región de la Araucanía, Chile	Support in counselling design	
María José	Arancibia			Universidad de La Frontera	Temuco, Región de la Araucanía, Chile	Phone follow-up support	
Irene	Stavros			Hospital de Antofagasta	Antofagasta, Región de Antofagasta, Chile	Recruitment support	
Francisca	Muñoz			Hospital de Antofagasta	Antofagasta, Región de Antofagasta, Chile	Intervention Support	
Yilia	Arias			Hospital de Antofagasta	Antofagasta, Región de Antofagasta, Chile	Measurement support	
Olga	Ferrada			Hospital de Antofagasta	Antofagasta, Región de Antofagasta, Chile	Intervention Support	
Camila	González			Hospital de Antofagasta	Antofagasta, Región de Antofagasta, Chile	Intervention Support	
Hanier	Verdejo			Hospital de Antofagasta	Antofagasta, Región de Antofagasta, Chile	Intervention Support	
María José	Segovia			Hospital de Antofagasta	Antofagasta, Región de Antofagasta, Chile	Intervention Support	
Karen	Adaros			Hospital de Antofagasta	Antofagasta, Región de Antofagasta, Chile	Recruitment support	
Camila	Aburto			Hospital de Antofagasta	Antofagasta, Región de Antofagasta, Chile	Intervention Support	
Fernando	Buzeta			Hospital de Antofagasta	Antofagasta, Región de Antofagasta, Chile	Intervention Support	
Evelyn	Valencia			Hospital Clínico Universidad de Chile	Santiago, Región Metropolitana, Chile	Measurement support	
José Tomás	Ramos			Hospital Clínico Universidad de Chile	Santiago, Región Metropolitana, Chile	Recruitment support	

Supplemental Online Content: Nonauthor Collaborators
*First name, last name, and suffix (if applicable) are required and will appear in PubMed.

***First Name and Middle Initial(s)**	***Last Name**	***Suffix (eg, Jr, III)**	Academic Degrees	Institution	Location (city, state/province, country)	Role or Contribution, eg, chair, principal investigator	Group (if more than 1 Group listed in the byline) and/or Subgroup (eg, Steering Committee)
Marcela	Osorio			Hospital Clínico Universidad de Chile	Santiago, Región Metropolitana, Chile	Intervention Support	
Fernanda	Díaz			Hospital Clínico Universidad de Chile	Santiago, Región Metropolitana, Chile	Measurement support	
Sylvia	Aravena			Hospital San Borja Arriarán	Santiago, Región Metropolitana, Chile	Intervention Support	
Silvia	Gatta			Hospital San Borja Arriarán	Santiago, Región Metropolitana, Chile	Recruitment support	
Juan Luis	Figueroa			Hospital San Borja Arriarán	Santiago, Región Metropolitana, Chile	Measurement support	
Daniela	Hermosilla			Hospital San Borja Arriarán	Santiago, Región Metropolitana, Chile	Intervention Support	
Iris	Díaz			Hospital San José	Santiago, Región Metropolitana, Chile	Measurement support	
Natalia	Maldonado			Hospital San José	Santiago, Región Metropolitana, Chile	Measurement support	
Rocío	Navarro-Rañinao			Hospital San José	Santiago, Región Metropolitana, Chile	Intervention Support	
Verónica	Olea			Hospital San Juan de Dios	Santiago, Región Metropolitana, Chile	Measurement support	
Ricardo	Aravena			Universidad de La Frontera	Temuco, Región de la Araucanía, Chile	Intervention Support	
Paulina	Sánchez			Universidad de La Frontera	Temuco, Región de la Araucanía, Chile	Recruitment support	
Kony	Morales			Universidad de La Frontera	Temuco, Región de la Araucanía, Chile	Measurement support	
Marilia	Saldias			Universidad de La Frontera	Temuco, Región de la Araucanía, Chile	Recruitment support	
María Francisca	Contreras			Universidad de La Frontera	Temuco, Región de la Araucanía, Chile	Phone follow-up support	
