# Supplementary material for: Hybrid Cardiac Rehabilitation Program in a Low-Resource Setting: A Randomized Clinical Trial
Source: JAMA Netw Open. 2024 Jan 9;7(1):e2350301. doi: 10.1001/jamanetworkopen.2023.50301 (PMC10777264; doi:10.1001/jamanetworkopen.2023.50301)
Supplement: Supplement 4. — Data Sharing Statement [file jamanetwopen-e2350301-s004.pdf]

Data Sharing Statement

Seron. Hybrid Cardiac Rehabilitation Program in a Low-Resource Setting. *JAMA Netw Open*. Published January 09, 2024. doi:10.1001/jamanetworkopen.2023.50301

Data
Data available: Yes
Data types: Deidentified participant data
How to access data: pamela.seron@ufrontera.cl
When available: With publication

Supporting Documents
Document types: None

Additional Information
Who can access the data: researchers whose proposed use of the data has been approved
Types of analyses: for metanalysis
Mechanisms of data availability: after approval of a proposal
